# Novel Genomic and Evolutionary Perspective of Cyanobacterial tRNAs

**DOI:** 10.3389/fgene.2017.00200

**Published:** 2017-12-13

**Authors:** Tapan K. Mohanta, Asad S. Syed, Fuad Ameen, Hanhong Bae

**Affiliations:** ^1^School of Biotechnology, Yeungnam University, Gyeongsan, South Korea; ^2^Department of Botany and Microbiology, College of Science, King Saud University, Riyadh, Saudi Arabia

**Keywords:** cyanobacteria, tRNA, evolution, intron, transition, transversion

## Abstract

Transfer RNA (tRNA) plays a central role in protein synthesis and acts as an adaptor molecule between an mRNA and an amino acid. A tRNA has an L-shaped clover leaf-like structure and contains an acceptor arm, D-arm, D-loop, anti-codon arm, anti-codon loop, variable loop, Ψ-arm and Ψ-loop. All of these arms and loops are important in protein translation. Here, we aimed to delineate the genomic architecture of these arms and loops in cyanobacterial tRNA. Studies from tRNA sequences from 61 cyanobacterial species showed that, except for few tRNAs (tRNA^Asn^, tRNA^Leu^, tRNA^Gln^, and tRNA^Met^), all contained a G nucleotide at the 1st position in the acceptor arm. tRNA^Leu^ and tRNA^Met^ did not contain any conserved nucleotides at the 1st position whereas tRNA^Asn^ and tRNA^Gln^ contained a conserved U^1^ nucleotide. In several tRNA families, the variable region also contained conserved nucleotides. Except for tRNA^Met^ and tRNA^Glu^, all other tRNAs contained a conserved A nucleotide at the 1st position in the D-loop. The Ψ-loop contained a conserved U^1^-U^2^-C^3^-x-A^5^-x-U^7^ sequence, except for tRNA^Gly^, tRNA^Ala^, tRNA^Val^, tRNA^Phe^, tRNA^Thr^, and tRNA^Gln^ in which the U^7^ nucleotide was not conserved. However, in tRNA^Asp^, the U^7^ nucleotide was substituted with a C^7^ nucleotide. Additionally, tRNA^Arg^, tRNA^Gly^, and tRNA^Lys^ of cyanobacteria contained a group I intron within the anti-codon loop region. Maximum composite likelihood study on the transition/transversion of cyanobacterial tRNA revealed that the rate of transition was higher than the rate of transversion. An evolutionary tree was constructed to understand the evolution of cyanobacterial tRNA and analyses revealed that cyanobacterial tRNA may have evolved polyphyletically with high rate of gene loss.

## Introduction

**Figure 1:**
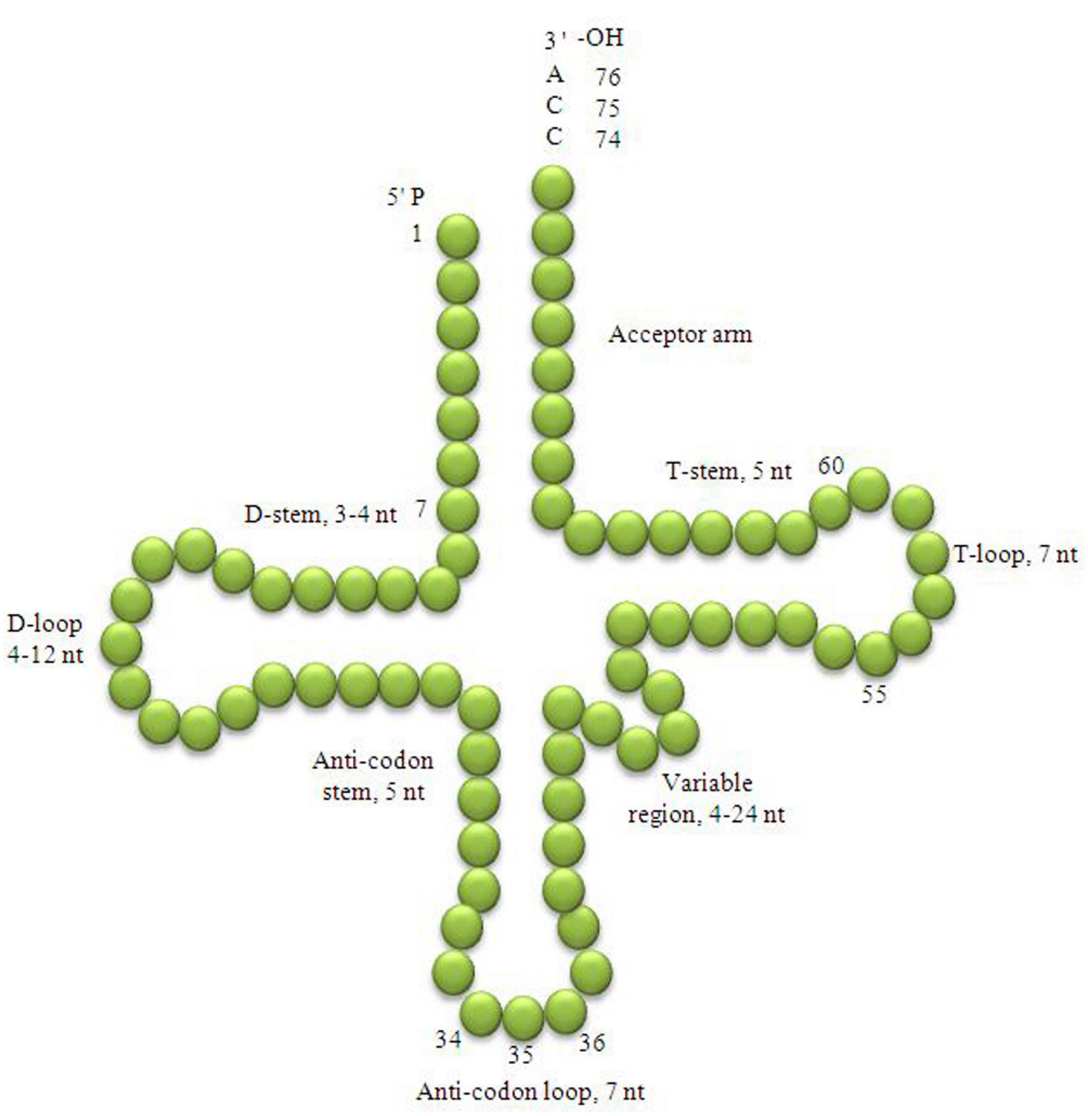
Clover leaf-like structure of tRNA. The tRNA possess the acceptor arm (7 nt), D-arm (3–4 nt), D-loop (4–12 nt), anti-codon arm (5 nt), anti-codon loop (7 nt), variable region (4–23 nt), Ψ-arm (5 nt) and Ψ-loop (7 nt). The D-arm, D-loop, and variable region possess variable number of nucleotides whereas the nucleotide number in the acceptor arm, anti-codon arm, anti-codon loop, Ψ-arm and Ψ-loop is always constant. The anti-codon nucleotides in the anti-codon loop is always numbered 34, 35, and 36 whereas, the nucleotides of C-C-A tail is always numbered with 74, 75, and 76.

Transfer RNAs (tRNA) are short non-coding RNAs comprised of 75–95 nucleotides, universally present in all organisms from the prokaryotes to the eukaryotes. The 75–95 residues polynucleotide sequences of tRNAs fold back upon themselves and form hydrogen-bonded clover leaf-like structures that fold into L-shaped tertiary structure (Holley et al., 1965; Wilusz, 2015). The tRNA is organized into a linear double stranded helix. Determination of the three-dimensional structure of a tRNA was a landmark discovery for modern molecular biology research. The major function of the tRNA is to bridge between an amino acid and an mRNA during protein synthesis where the tRNA transfers the cognate amino acid to the translating polypeptide chain. The clover leaf-like structure of the tRNA is comprised of an acceptor arm, D-arm, D-loop, anti-codon arm, anti-codon loop, variable loop, Ψ-arm, and Ψ-loop (Figure 1; Kirchner and Ignatova, 2014; Mohanta and Bae, 2017). The acceptor arm is 7 base pairs long, the D-arm is 3–4 base pairs, the D-loop is 4–12 nucleotides, the anti-codon arm is 5 base pairs, the anti-codon loop is 7 nucleotides, the variable loop is 4–23 nucleotides, the Ψ-arm is 5 base pairs and the Ψ-loop is 7 nucleotides long (Figure 1; Kirchner and Ignatova, 2014). The heterogeneity in the length of tRNAs is caused by variable number of bases in D-loop and the variable loop. The most important functional parts of a tRNA are: the anti-codon triplet, which reads the codons of a messenger RNA; a 3′-CCA tail where the cognate amino acid is attached and the Ψ-arm and Ψ-loop that hold the ribosome machinery.

There are 21 different types of iso-acceptor families for the 20 amino acids, one for each amino acid and one for selenocysteine. In prokaryotes, an isoacceptor family may have one (tRNA^Trp^, and tRNA^Met^) to six tRNA members (tRNA^Ser^, tRNA^Arg^, and tRNA^Leu^). To date, four different types of tRNA genes have been reported so far including intron-containing, non-intronic, split and permutated types (Randau et al., 2005; Fujishima et al., 2009; Chan et al., 2011). The intron-containing and non-intronic tRNAs are encoded in a single gene, whereas the split tRNAs are encoded in two or more separate genes; in permutated tRNAs, the 3′ half of the tRNA is located up-stream to the 5′ half of the tRNA (Tocchini-Valentini et al., 2009; Maruyama et al., 2010; Soma, 2014). The intron-containing, permutated and split tRNA genes are called “disrupted tRNA genes” whereas the non-intronic tRNA genes are called “continuous tRNA genes” (Sugahara et al., 2009; Kanai, 2015). The disrupted tRNA genes may have evolved earlier than the continuous genes (Di Giulio, 2008a,b; Fujishima et al., 2009). The tRNA mini helix is the most ancient form of tRNA and forms the top half of the tRNA that contains the acceptor arm and the Ψ-arm (Weiner and Maizels, 1987, 1999; Shi et al., 1992, 1998; Sun and Caetano-Anollés, 2008). The tRNA mini-helix is a validated substrate for aminoacyl-tRNA synthetases and the 3′-CCA tail adding enzymes (Shi et al., 1992, 1998). The genomic tag hypothesis suggests that the upper half of the tRNA had evolved earlier than the lower half. Phylogenetic analysis also suggests that the top half of the tRNA is more ancient than the bottom half (Sun and Caetano-Anollés, 2008). The evolution of tRNA occurred with respect to its surrounding environment and is far more complex than that of proteins, because tRNA perform multiple roles in the cell (Navarre and Schneewind, 1999; Kirchner and Ignatova, 2014). tRNA interacts with numerous molecules; during protein synthesis, it interacts with amino-acyl tRNA synthetase, ribosome and messenger RNA, and a large number of transcription factors and other enzymes (Nirenberg and Leder, 1964). tRNA^Gly^ acts as a structural element of the peptidoglycan of the bacterial cell wall whereas tRNA^Lys^ and tRNA^Ala^ are used to aminoacylate membrane lipid to permeate cationic antibiotics (Roy and Ibba, 2008). Cyanobacteria are the most ancient living organism whose existence dates back to 3.3–3.5 billion years (Schopf and Packer, 1987; Altermann et al., 2006). Because it is most likely that the tRNA genes date back to the age of cyanobacteria, and studying the genomic and evolutionary perspectives of cyanobacterial tRNA is very important. Given their high level of conservation it is especially important to study the genomics, origin and evolution of tRNAs. Here, a comprehensive study of cyanobacterial tRNA was conducted by analyzing the tRNA sequences of 61 cyanobacterial species.

## Materials and methods

### Identification and analysis of cyanobacterial tRNAs

The genomic tRNA sequences of cyanobacteria were searched from Joint Genome Portal (http://genome.jgi.doe.gov/) and cyanobacterial genome database of National Center for Biotechnology Information (https://www.ncbi.nlm.nih.gov/). In total, tRNA sequences from 61 cyanobacterial species were retrieved and analyzed (Supplementary data 1). The downloaded genomic tRNA sequences of cyanobacterial species were analyzed using tRNAScan-SE software (Lowe and Eddy, 1997). All the sequences analyzed during this study were found to encode for tRNA. Different statistical parameters used to run the tRNAScan-SE software were as follows: sequence source, bacterial/mixed; search mode, default; and genetic code for tRNA isotype prediction, universal. The tRNAScan-SE software is the most accurate software and it identifies 99–100% tRNA genes from DNA sequences. It produces less than one false positive per 15 gigabases (Lowe and Eddy, 1997).

### Sequence alignment

Multiple sequence alignment was conducted to identify the conserved consensus sequences of cyanobacterial tRNA. Multalin software was used to conduct the multiple sequence alignment of cyanobacterial tRNA using the default parameters as described previously (Mohanta et al., 2015a,b,c, 2016) with minor modification.

### Analysis of transition/transversion rate of cyanobacterial tRNAs

To analyze the transition and transversion rate of the cyanobacterial tRNA, a clustal file for each of the tRNA gene family was generated using the multiple sequence alignment program MUSCLE (http://www.ebi.ac.uk/Tools/msa/muscle/). The generated clustal file was downloaded and converted to a MEGA file format. The generated MEGA file of the cyanobacterial tRNA was uploaded in MEGA6 software to analyze the transition/transversion rate (Tamura et al., 2013). Following statistical parameters were used to analyze the transition/transversion rate: analysis, estimate transition/transversion bias (MCL); scope, all selected taxa; statistical method, maximum composite likelihood; substitution type, nucleotide; model/method, Tamura-Nei model; and gaps/missing data treatment, pair wise deletion.

### Construction of phylogenetic trees

A phylogenetic tree was constructed to understand the evolutionary aspects of cyanobacterial tRNAs. All the genomic tRNA sequences of cyanobacterial tRNA were subjected to clustal omega server to construct a clustal file. Generated clustal file of the cyanobacterial tRNA was converted to MEGA file format using MEGA6 software. The MEGA files of the cyanobacterial tRNA were subjected to MEGA6 software to construct the phylogenetic trees (Tamura et al., 2013). Following statistical parameters were used to construct the phylogenetic tree: analysis, phylogeny reconstruction; scope, all selected taxa; statistical method, neighbor-joining; test of phylogeny, bootstrap method; no. of bootstrap replicate, 1,000; substitution type, nucleotide; model/method, maximum composite likelihood; substitution to include, d: transition+transversion; rates among sites, uniform rates; pattern among lineage, same (homogeneous); and gaps/missing data treatment, pair wise deletion. The pairwise distances and substitution model parameters were estimated by maximizing the composite likelihood because maximum likelihood approach can accurately estimate the parameters that drive the evolutionary process (Akaike, 1998; Varin et al., 2011; Xu and Reid, 2011; Zhao et al., 2015). Phylogenetic trees with low bootstrap values were collapsed with 50% cutoff values.

### Gene duplication and loss

To understand the duplication and loss event of cyanobacterial tRNA, all the clustal files generated to study the transition/transversion rate of individual tRNA family were used to construct the phytlogenetic tree. Twenty phylogenetic trees were generated separately for each tRNA gene family using the similar statistical parameters as mentioned previously. A species tree of studied cyanobacterial species was downloaded from NCBI (https://www.ncbi.nlm.nih.gov/Taxonomy/CommonTree/wwwcmt.cgi). The phylogenetic tree of the cyanobacterial tRNA and species tree of cyanobacterial species were subjected to Notung2.6 software and further reconciliation of gene tree with the species tree led to finding of duplicated and loss genes of cyanobacterial tRNA (Chen et al., 2000; Vernot et al., 2008).

## Results

### The conservation of tRNA sequences are family specific

To understand the basic genomic and evolutionary aspects of cyanobacterial tRNAs, genomic tRNA sequences of cyanobacterial species were downloaded from the Joint Genome Institute (JGI) genome portal and National Center for Biotechnology Information (NCBI) (Table 1). In total, the tRNA sequences of 61 cyanobacterial species were downloaded and analyzed for their conserved genomic aspects. A conserved genomic sequence is required for the conserved clover leaf-like structure of a tRNA. Although significant conservation is present in tRNAs at the nucleotide level, occasionally the conserved structure of tRNAs varies at the family level.

**Table 1:** Cyanobacterial species and the number of tRNA genes used during this study.

**Sl. No**.	**Name of the species**	**No. of tRNAs**
1	*Anabaena cylindrica* PCC 7122	61
2	*Anabaena* sp. PCC 7108	43
3	*Calothrix desertica* PCC 7102	65
4	*Calothrix* sp. PCC 6303	40
5	*Calothrix* sp. PCC 7103	65
6	*Calothrix* sp. PCC 7507	72
7	*Chamaesiphon minutus* PCC 6605	54
8	*Cyanobacterium* PCC 7702	49
9	*Chroococcidiopsis* sp. PCC 6712	51
10	*Chroococcidiopsis thermalis* PCC 7203	46
11	*Crinalium epipsammum* PCC 9333	43
12	*Cyanobacterium cyanothece* sp. BH63E	42
13	*Cyanobacterium* ESFC-1	72
14	*Cyanobacterium aponinum* PCC 10605	43
15	*Cyanobacterium stanieri* PCC 7202	43
16	*Cyanobium gracile* PCC 6307	44
17	*Cylindrospermum stagnale* PCC 7417	69
18	*Dactylococcopsis salina* PCC 8305	42
19	*Fischerella* sp. PCC 9339	50
20	*Fischerella* sp. PCC 9431	69
21	*Fischerella* sp. PCC 9605	42
22	*Geitlerinema* sp. PCC 7105	50
23	*Geitlerinema* sp. PCC 7407	46
24	*Geminocystis herdmanii* PCC 6308	40
25	*Gloecapsa* sp. PCC 73106	40
26	*Gloecapsa* sp. 7428	41
27	*Halothece* sp. PCC 7418	46
28	*Kamptonema formosum*	71
29	*Leptolyngbya boryana* PCC 6306	67
30	*Leptolyngbya* sp. PCC 6406	44
31	*Leptolyngbya* sp. PCC 7375	63
32	*Leptolyngbya* sp. PCC 7376	44
33	*Mastigocladopsis repens* PCC 10914	43
34	*Microchaete* sp. PCC 7126	69
35	*Microcoleus* sp. PCC 7113	70
36	*Microcoleus vaginatus* FGP-2	72
37	*Nodosilinea nodulosa* PCC 7104	43
38	*Nostoc* sp. PCC 7107	78
39	*Nostoc* sp. PCC 7524	65
40	*Oscillatoria acuminata* PCC 6304	66
41	*Oscillatoria* sp. PCC 10802	65
42	*Oscillatoria nigro-viridis* PCC 7112	71
43	*Oscillatoriales* sp. JSC-12	44
44	*Pleurocapsa* sp. PCC 7319	42
45	*Pleurocapsa* sp. PCC 7327	42
46	*Prochlorothrix hollandica* PCC 9006	41
47	*Pseudanabaena* sp. PCC 6802	75
48	*Pseudanabaena* sp. PCC 7367	46
49	*Pseudanabaena* sp. PCC 7429	44
50	*Rivularia* sp. PCC 7116	52
51	*Scytonema hofmannii* UTEX 2349	53
52	*Spirulina major* PCC 6313	43
53	*Spirulina subsalsa* PCC 9445	41
54	*Stanieria cyanosphaera* PCC 7437	43
55	*Synechococcus elongatus* PCC 7942	44
56	*Synechococcus* sp. PCC 9616	42
57	*Synechococcus* sp. PCC 6312	41
58	*Synechococcus* sp. PCC 7336	44
59	*Synechococcus* sp. PCC 7502	43
60	*Synechocystis* sp. PCC 7509	46
61	*Xenococcus* sp. PCC 7305	36

In tRNA^Gly^ of the *Pseudanabaena* sp. PCC 7367 (gene id: 2504679288) was found to contain only a conserved C^2^ nucleotide instead of the conserved G^1^-C^2^-G^3^ (Table 2, Supplementary Figure 1). Similarly, the acceptor arm of *Cylindrospermum stagnale* PCC 7417 (gene id: 2509767604) was found to contain G^1^-G^2^-A^3^ nucleotides. The nucleotide U^8^ and A^9^ in the acceptor arm of tRNA^Gly^ was found to be conserved (Table 3). The D-arm was found to be unconserved whereas the D-loop has conserved A^1^-x_2_-G^4^-G^5^ nucleotides followed by the presence of conserved U^2^-x-C^4^-C^5^-A^6^ nucleotides in anti-codon arm (Table 2). The variable loop was found to be unconserved whereas the Ψ-arm has conserved G^5^ nucleotide and the Ψ-loop has conserved U^1^-U^2^-C^3^-x-A^5^ nucleotides (Table 2).

**Table 2:** Conserved nucleotide signature elements of Cyanobacterial tRNAs.

**tRNA Isotypes**	**5′ Acceptor Arm (1–7 nt)**	**D-arm (10–13 nt)**	**D-loop**	**AC- arm (27–31 nt)**	**Anti-codon loop (32–38 nt)**	**Variable loop**	**Ψ-arm (49–53 nt)**	**Ψ-loop (54–60 nt)**
Gly	G^1^-C^2^-G^3^	^*****^	A^1^-x_2_-G^4^-G^5^	^*****^	U^2^-x-C^4^-C^5^-A^6^	^*****^	G^5^	U^1^-U^2^-C^3^-x-A^5^
Ala	G^1^-G^2^-G^3^	G^1^-C^2^-U^3^-C^4^	A^1^-x_2_-U^4^-G^5^-G^6^-x-A^8^	^*****^	U^2^-x-G^4^-C^5^-A^6^	G^5^	G^5^	U^1^-U^2^-C^3^-x-A^5^
Pro	C^1^-x-G^3^-G^4^-x_2_-G^7^	C^4^	A^1^-G^2^-x_6_-A^9^	^*****^	U^1^-U^2^-x-G^4^-G^5^-G^6^	G^3^-x-C^5^	G^1^-x_2_-G^4^-G^5^	U^1^-U^2^-C^3^-x-A^5^-A^6^-U^7^
Val	G^1^-G^2^-x-C^4^	C^2^-x-C^4^	A^1^-G^2^-x_3_-G^6^-x-U^8^-A^9^	^*****^	U^1^-U^2^-x-A^4^-C^5^-A^6^	G^3^-U^4^-C^5^	G^4^-G^5^	U^1^-U^2^-C^3^-x-A^5^
Leu	^*****^	^*****^	A^1^-A^2^-x_2_-G^5^-G^6^-x-A^8^	^*****^	U^2^-x-A^4^	^*****^	G^5^	U^1^-U^2^-C^3^-x-A^5^-x-U^7^
Ile	G^1^-G^2^-G^3^-C^4^	G^1^-C^2^-U^3^-C^4^	A^1^-x_4_-G^6^-x_2_-A^9^	^*****^	C^1^-U^2^-x-A^4^-U^5^-A^6^-A^7^	G^3^-U^4^	G^4^-G^5^	U^1^-U^2^-C^3^-x-A^5^-x-U^7^
Met	^*****^	G^2^	G^6^	^*****^	C^1^-U^2^-C^3^-A^4^-U^5^-A^6^-A^7^	^*****^	G^5^	U^1^-U^2^-C^3^-x-A^5^-x-U^7^
Phe	C^1^/G^1^-C^2^-C^3^-x_2_-G^6^	G^1^-C^2^-U^3^-C^4^	A^1^-G^2^-U^3^-U^4^-G^5^-G^6^-U^7^	G^4^	U^2^-G^3^-A^4^-A^5^-x-A^7^	G^3^-U^4^-C^5^	G^5^	U^1^-U^2^-C^3^-x-A^5^
Tyr	G^1^-G^2^-G^3^-U^4^-C^5^	G^1^-x-C^3^-C^4^	A^1^-G^2^-U^3^-G^4^-G^5^-U^6^-U^7^-A^8^	^*****^	U^2^-G^3^-U^4^-A^5^	^******^	G^4^-G^5^	U^1^-U^2^-C^3^-x-A^5^-x-U^7^
Trp	G^1^	G^1^-U^2^	A^1^-x_3_-G^5^	G^4^-U^5^	C^1^-U^2^-C^3^-C^4^-A^5^-A^6^-A^7^	^******^	G^5^	U^1^-U^2^-C^3^-x-A^5^-x-U^7^
Ser	G^1^-G^2^-A^3^	G^1^-C^2^	A^2^-x_2_- G^5^- G^6^	^*****^	U^2^-x_3_-A^5^-A^6^	^******^	A^4^/G^4^-G^5^	U^1^-U^2^-C^3^-x-A^5^-x-U^7^
Thr	G^1^-C^2^	G^1^-C^2^-x-C^4^	U^2^-x-G^4^-U^5^-A^6^-A^7^	^*****^	U^2^-x-G^4^-U^5^-A^6^-A^7^	G^3^	G^5^	U^1^-U^2^-C^3^-x-A^5^
Cys	G^1^	G^1^-C^2^-C^3^	A^1^-A^2^-G^3^-x-G^5^- G^6^-U^7^	^*****^	C^1^-U^2^-G^3^-C^4^-A^5^-A^6^-A^7^	C^5^	G^5^	U^1^-U^2^-C^3^-x-A^5^-x-U^7^
Asn	U^1^-C^2^-C^3^-x-C^5^	G^1^-C^2^-U^3^	A^1^-x_1−2_-G^3^-G^4^	^*****^	C^1^-U^2^-G^3^-U^4^-U^5^-A^6^-A^7^	G^3^-U^4^	G^5^	U^1^-U^2^-C^3^-x-A^5^-x-U^7^
Gln	U^1^-G^2^-x_3−4_-G^6^	^****^	A^1^/U^1^-A^2^-x_2_-G^4^	^*****^	U^1^-U^2^-U^3^-U^4^-G^5^	^*****^	A^3^-G^4^-G^5^	U^1^-U^2^-C^3^-x-A^5^
Lys	G^1^-G^2^-G^3^	C^2^	A^1^-x_2_- G^4^-G^5^	^*****^	C^1^-U^2^-U^3^-U^4^-U^5^-A^6^-A^7^	^*****^	G^4^-G^5^	U^1^-U^2^-C^3^-x-A^5^-x-U^7^
His	G^1^-C^2^/U^2^-G^3^-x_3_-G^7^	G^1^-C^2^-C^3^	A^1^-A^2^-G^3^-x- G^5^- G^6^-U^7^	G^4^-G^5^	U^1^-U^2^-G^3^-U^4^-G^5^	^*****^	G^1^-x-G^3^-G^4^-G^5^	U^1^-U^2^-C^3^-x-A^5^-x-U^7^
Arg	G^1^-G^2^	G^1^	A^1^-x_2−3_-G^4^-x_3_-A^8^	^****^	U^1^/ C^1^-U^2^-x-C^4^	^*****^	G^4^-G^5^	U^1^-U^2^-C^3^-x-A^5^-x-U^7^
Asp	G^1^-G^2^-G^3^	G^1^-x-U^3^-C^4^	A^1^-x_2−3_-G^4^- G^5^-U^6^-x-A^8^	^****^	C^1^-U^2^-G^3^-U^4^-C^5^-A^6^	A^1^-x-G^3^-U^4^	G^1^-x-G^3^-x-G^5^	U^1^-U^2^-C^3^-x-A^5^-x-C^7^
Glu	G^1^	^****^	G^5^	^****^	U^2^-U^3^-U^4^-C^5^	^****^	G^3^-x-G^5^	U^1^-U^2^-C^3^-x-A^5^-x-U^7^

The asterisk (^*^) mark represent the absence of conserved nucleotide consensus sequence in respective region of the tRNA.

**Table 3:** Variations of nucleotide conservation at the 8th and 9th position of tRNAs in the 5′ end of the acceptor arm in cyanobacteria.

**tRNA**	**8th position**	**9th position**
Glycine (Gly)	U	A
Alanine (Ala)	U	A
Proline (Pro)	U	A
Valine (Val)	U	A
Leucine (Leu)	U	G
Isoleucine (Ile)	U	A
Methionine (Met)	U	A/G/U
Phenylalanine (Phe)	U	A
Tyrosine (Tyr)	U	G
Tryptophan (Trp)	U	A
Serine (Ser)	U	G
Threonine (Thr)	U	A/G
Cysteine (Cys)	U	A/C/G
Asparagine (Asn)	U	A
Glutamine (Gln)	U	A/C/G
Lysine (Lys)	U	A/G
Histidine (His)	U/A	A/C
Arginine (Arg)	U	A/G
Aspartate (Asp)	U	A
Glutamate (Glu)	U/C/G	A/C/U

In tRNA^Ala^, the acceptor arm was found to contain conserved G^1^-G^2^-G^3^ nucleotides at the 5′end (Table 2) whereas tRNA^Ala^ of *Pseudanabaena* sp. PCC 6802 (gene id: 2507088349) and *Microchaete* sp. PCC 7126 (gene id: 2509784201) was found to contain A^1^-G^2^-G^3^ nucleotides. The nucleotide U^8^ and A^9^ in the acceptor arm were found to be conserved (Table 3) followed by the presence of conserved G^1^-C^2^-U^3^-C^4^ nucleotides in the D-arm. The D-loop contained conserved A^1^-x_2_-U^4^-G^5^-G^6^-x-A^8^ nucleotides followed by the presence of conserved U^2^-x-G^4^-C^5^-A^6^ nucleotides in the anti-codon arm. A G^5^ nucleotide was found to be conserved in the variable region as well as in the Ψ-arm followed by the presence of conserved U^1^-U^2^-C^3^-x-A^5^ nucleotides in the Ψ- loop (Table 2).

The acceptor arm of tRNA^Pro^ was found to contain conserved C^1^-x-G^3^-G^4^-x_2_-G^7^ nucleotides (Table 2). The nucleotide U^8^ and A^9^ in the acceptor arm and C^4^ in the D-arm were found to be conserved followed by the presence of conserved A^1^-G^2^-x_6_-A^9^ nucleotides in the D-loop (Tables 2, 3). The anti-codon loop contained conserved U^1^-U^2^-x-G^4^-G^5^-G^6^ followed by the presence of conserved G^3^-x-C^5^ nucleotides in the variable region. The Ψ-arm contained conserved G^1^-x_2_-G^4^-G^5^ nucleotides followed by the presence of conserved U^1^-U^2^-C^3^-x-A^5^-A^6^-U^7^ nucleotides in the Ψ- loop (Table 2).

The acceptor arm of tRNA^Val^ was found to contain conserved G^1^-G^2^-x-C^4^ nucleotides (Table 2) except for species *Cyanobacterium* sp. ESFC 1 (gene id: 2517646710), *Microcoleus vaginatus* FGP-2 (gene id: 2506348931), *Nostoc* sp. PCC 7107 (gene id: 2503741640), *Kamptonema formosum* PCC 6407 (gene id: 2508873173), *Pseudanabaena* sp. PCC 6802 (gene id: 2507089028), *Oscillatoria acuminate* PCC 6304 (gene id: 2509421383), *Microchaete* sp.PCC 7126 (gene id: 2509784199), and *Microcoleus* sp. PCC 7113 (gene id: 2509433894). The tRNA^Val^ of *Pleurocapsa* sp. PCC 7319 (gene id: 2509708986). They were found to contain U^1^ nucleotide instead of the G^1^ or A^1^ nucleotides. The U^8^ and A^9^ nucleotides were conserved in tRNA^Val^ (Table 3) followed by the presence of conserved C^2^-x-C^4^ nucleotides in the D-arm. The D-loop contained conserved A^1^-G^2^-x_3_-G^6^-x-U^8^-A^9^ nucleotides followed by the presence of conserved U^1^-U^2^-x-A^4^-C^5^-A^6^ nucleotides in the anti-codon arm (Table 2). The variable region contained conserved G^3^-U^4^-C^5^ nucleotides, followed by the presence of conserved G^4^-G^5^ nucleotides in the Ψ-arm and U^1^-U^2^-C^3^-x-A^5^ nucleotides in the Ψ- loop (Table 2).

The acceptor arm of tRNA^Leu^ contained variable nucleotides. However, more than half of the tRNA^Leu^ were contained G^1^-C^2^-G^3^ nucleotides (Table 2). The U^8^ and U^9^ nucleotide were conserved at the 8th and 9th position, respectively (Table 3), whereas the D-arm did not contain any conserved nucleotide sequences whereas the D-loop contained conserved A^1^-A^2^-x_2_-G^5^-G^6^-x-A^8^ nucleotides. The anti-codon loop contained conserved U^2^-x-A^4^ nucleotides followed by the presence of G^5^ nucleotide in the Ψ-arm and U^1^-U^2^-C^3^-x-A^5^-x-U^7^ nucleotides in the Ψ- loop (Table 2). No conserved nucleotides were found in the variable loop.

The acceptor arm of tRNA^Ile^ contained conserved G^1^-G^2^-G^3^-C^4^ nucleotides (Table 2). The nucleotides U^8^ and A^9^ were also found to be conserved in their respective position (Table 3) followed by the presence of conserved G^1^-C^2^-U^3^-C^4^ nucleotides in the D-arm and A^1^-x_4_-G^6^-x_2_-A^9^ nucleotides in the D-loop. The anti-codon loop contained conserved C^1^-U^2^-x-A^4^-U^5^-A^6^-A^7^ nucleotides followed by the presence of conserved G^3^-U^4^ nucleotides in the variable region, G^4^-G^5^ nucleotides in the Ψ-arm and U^1^-U^2^-C^3^-x-A^5^-x-U^7^ nucleotides in the Ψ-loop (Table 2).

In tRNA^Met^, no consensus conserved nucleotides were found at the 5′ end of the acceptor arm. A few tRNAs were contained G^1^-G^2^-C^3^ nucleotides whereas some other were contained C^1^-G^2^-C^3^, or C^1^-C^2^-A^3^ nucleotides (Table 2). The U nucleotide at the 8th position was conserved in the acceptor arm whereas the 9th position was occupied by either A^9^/G^9^ or U^9^ nucleotide. The D-arm and the D-loop was found to contain conserved G^2^ and G^6^ nucleotide, respectively. The anti-codon loop contained conserved C^1^-U^2^-C^3^-A^4^-U^5^-A^6^-A^7^ nucleotides followed by the presence of conserved G^5^ nucleotide in the Ψ-arm and U^1^-U^2^-C^3^-x-A^5^-x-U^7^ nucleotides in the Ψ-loop (Table 2).

The acceptor arm of tRNA^Phe^ contained conserved C^1^/G^1^-C^2^-C^3^-x_2_-G^6^ nucleotides (Table 2). The nucleotides U^8^ and A^9^ in the acceptor arm were conserved (Table 3) followed by the presence of conserved G^1^-C^2^-U^3^-C^4^ nucleotides in the D-arm and A^1^-G^2^-U^3^-U^4^-G^5^-G^6^-U^7^ nucleotides in the D-loop, respectively. In the majority of the cases, the anti-codon arm contained conserved G^4^ nucleotide and the anti-codon loop contained conserved U^2^-G^3^-A^4^-A^5^-x-A^7^ nucleotides. The variable loop contained conserved G^3^-U^4^-C^5^ nucleotides followed by the presence of conserved G^5^ nucleotide the Ψ-arm and U^1^-U^2^-C^3^-x-A^5^ nucleotides in the Ψ-loop (Table 2).

In tRNA^Tyr^, the acceptor arm contained conserved G^1^-G^2^-G^3^-U^4^-C^5^ nucleotides (Table 2). The U^8^ and G^9^ nucleotides in the acceptor arm were conserved (Table 3) followed by the presence of conserved G^1^-x-C^3^-C^4^ nucleotides in the D-arm and A^1^-G^2^-U^3^-G^4^-G^5^-U^6^-U^7^-A^8^ nucleotides in the D-loop, respectively (Table 2). The anti-codon loop contained conserved U^2^-G^3^-U^4^-A^5^ nucleotides followed by the presence of conserved G^4^-G^5^ nucleotides in the Ψ-arm and U^1^-U^2^-C^3^-x-A^5^-x-U^7^ nucleotides in the Ψ-loop (Table 2).

In the majorities of cases, the acceptor arm of tRNA^Trp^ contained a conserved G^1^ nucleotide whereas few others were found to contain A^1^ nucleotide. The U^8^ nucleotide of the acceptor arm was conserved whereas A^9^ nucleotide was sometimes substituted with G^9^ nucleotide (Tables 2, 3). The D-arm contained conserved G^1^-U^2^ nucleotides followed by the presence of conserved A^1^-x_3_-G^5^ nucleotides in the D-loop. The anti-codon arm contained conserved G^4^-U^5^ nucleotides followed by the presence of conserved C^1^-U^2^-C^3^-C^4^-A^5^-A^6^-A^7^ nucleotides in the anti-codon loop, a G^5^ nucleotide in the Ψ-arm and U^1^-U^2^-C^3^-x-A^5^-x-U^7^ nucleotides in the Ψ-loop, respectively (Table 2).

In tRNA^Ser^, the acceptor arm contained conserved G^1^-G^2^-A^3^ nucleotides (Table 2). A few of the tRNA^Ser^ were contained U^1^ or G^1^ nucleotides instead of G^1^-G^2^-A^3^ consensus nucleotide sequence. The U^8^ and G^9^ nucleotide were found to be conserved (Table 3) followed by the presence of conserved G^1^-C^2^ nucleotides in the D-arm and A^2^-x_2_-G^5^-G^6^ nucleotides in the D-loop, respectively. The anti-codon loop contained conserved U^2^-x_3_-A^5^-A^6^ nucleotides (Table 2) followed by the presence of conserved C^21^ nucleotide in variable region, A^4^/G^4^-G^5^ nucleotides in the Ψ-arm and U^1^-U^2^-C^3^-x-A^5^-x-U^7^ nucleotides in the Ψ-loop, respectively (Table 2).

The acceptor arm of tRNA^Thr^ contained conserved G^1^-C^2^ nucleotides (Table 2). The U^8^ nucleotide was found to be conserved whereas the 9th position was occupied by A^9^ or G^9^ nucleotide (Table 3). The D-arm contained conserved G^1^-C^2^-x-C^4^ nucleotides and A^1^-x_1−4_-G^3^-G^4^-x-A^6^ nucleotides in the D-loop, respectively. The anti-codon loop contained conserved U^2^-x-G^4^-U^5^-A^6^-A^7^ nucleotides followed by the presence of conserved G^3^ nucleotide in the variable loop, G^5^ nucleotide in the Ψ-arm and U^1^-U^2^-C^3^-x-A^5^ nucleotides in the Ψ-loop, respectively (Table 2). At the 7th position of the Ψ-loop, the U^7^ nucleotide was sometimes substituted with C^7^ nucleotide.

The acceptor arm of tRNA^Cys^ contained a conserved G^1^ nucleotide. The U^8^ and A^9^ nucleotides were found to be conserved while in some cases, A^9^ nucleotide was substituted with either C^9^ or G^9^ nucleotides (Table 3). The D-arm contained conserved G^1^-C^2^-C^3^ nucleotides followed by the presence of conserved A^1^-A^2^-G^3^-x-G^5^-G^6^-U^7^ and C^1^-U^2^-G^3^-C^4^-A^5^-A^6^-A^7^ nucleotides in the D-loop and anti-codon loop, respectively. The C^5^ nucleotide was conserved in the variable region of the tRNA^Cys^ followed by the presence of conserved G^5^ and U^1^-U^2^-C^3^-x-A^5^-x-U^7^ nucleotides in the Ψ-arm and Ψ-loop, respectively (Table 2).

The acceptor arm of tRNA^Asn^ contained conserved U^1^-C^2^-C^3^-x-C^5^ nucleotides (Table 2) with a few exceptions. The U^8^ and A^9^ nucleotides of the acceptor arm were conserved (Table 3) followed by the presence of conserved G^1^-C^2^-U^3^ nucleotides in the D-arm and A^1^-x_1−2_-G^3^-G^4^ nucleotides in the D-loop, respectively (Table 2). The anti-codon loop contained conserved C^1^-U^2^-G^3^-U^4^-U^5^-A^6^-A^7^ nucleotides followed by the presence of conserved G^3^-U^4^ nucleotides in the variable region, G^5^ in the Ψ-arm and U^1^-U^2^-C^3^-x-A^5^-x-U^7^ nucleotides in the Ψ-loop, respectively (Table 2).

The acceptor arm of tRNA^Gln^ contained conserved U^1^-G^2^-x_3−4_-G^6^ nucleotides (Table 2). The U^8^ nucleotide of the acceptor arm was conserved whereas the 9th position was substituted with either A^9^/C^9^, or G^9^ nucleotides (Table 3). The D-loop contained conserved A^1^/U^1^-A^2^-x_2_-G^4^ nucleotides followed by the presence of conserved U^1^-U^2^-U^3^-U^4^-G^5^ nucleotides in the anti-codon loop, A^3^-G^4^-G^5^ nucleotides in the Ψ-arm and U^1^-U^2^-C^3^-x-A^5^ nucleotides in the Ψ-loop, respectively (Table 2).

The acceptor arm at tRNA^Lys^ contained conserved G^1^-G^2^-G^3^ nucleotides (Table 2). In a few cases, the G^1^ nucleotide at the 1st position was substituted with either C^1^ or U^1^ nucleotide. The nucleotide U^8^ and A^9^ in the acceptor arm were conserved (Table 3) followed by the presence of conserved C^2^ nucleotide in the D-arm and A^1^-x_2_-G^4^-G^5^ nucleotides in the D-loop. The anti-codon loop contained conserved C^1^-U^2^-U^3^-U^4^-U^5^-A^6^-A^7^ nucleotides, followed by the presence of conserved G^4^-G^5^ sequence in the Ψ-arm and U^1^-U^2^-C^3^-x-A^5^-x-U^7^ nucleotides in the Ψ-loop (Table 2). In a few cases, the U^7^ nucleotide in the Ψ-loop was substituted with C^7^ nucleotide (Table 2).

In tRNA^His^, the acceptor arm contained conserved G^1^-C^2^/U^2^-G^3^-x_3_-G^7^ nucleotides with a few exceptions (Table 2). The U^8^ and A^9^ nucleotides in the acceptor arm were conserved followed by the presence of conserved G^1^-C^2^-C^3^ nucleotides in the D-arm and A^1^-A^2^-G^3^-x-G^5^-G^6^-U^7^ nucleotides in the D-loop, respectively (Table 2). The anti-codon arm contained conserved G^4^-G^5^ nucleotides followed by the presence of conserved U^1^-U^2^-G^3^-U^4^-G^5^ nucleotides in the anti-codon loop, G^1^-x-G^3^-G^4^-G^5^ nucleotides in the Ψ-arm and U^1^-U^2^-C^3^-x-A^5^-x-U^7^nucleotides in the Ψ-loop. In a few cases, the U^7^ nucleotide in the Ψ-loop was substituted with C^7^ nucleotide (Table 2).

In tRNA^Arg^, the acceptor arm at the 5′ end contained conserved G^1^-G^2^ nucleotides (Table 2). In a few cases, the G^1^ nucleotide at the 1st position substituted by A^1^ or C^1^ nucleotide. The U^8^ nucleotide in the acceptor arm was conserved whereas the 9th position was substitutes with A^9^/G^9^ or C^9^ nucleotides (Table 3). The D-arm was found to contain conserved G^1^ nucleotide, followed by the presence of conserved A^1^-x_2−3_-G^4^-x_3_-A^8^ nucleotide in the D-loop and C^1^/U^1^-U^2^-x-C^4^ nucleotides in the anti-codon loop (Table 2). The Ψ-arm and Ψ-loop was found to contain conserved G^4^-G^5^ and U^1^-U^2^-C^3^-x-A^5^-x-U^7^ nucleotides, respectively (Table 2).

The acceptor arm of tRNA^Asp^ contained conserved G^1^-G^2^-G^3^ nucleotides (Table 2). A few species were found to contain C^1^ nucleotide instead of G^1^ nucleotide at the 1st position. The U^8^ and A^9^ nucleotides in the acceptor arm were conserved (Table 3), followed by the presence of conserved G^1^-x-U^3^-C^4^ and A^1^-x_2−3_-G^4^-G^5^-U^6^-x-A^8^ nucleotides in the D-arm and D-loop, respectively (Table 2). The anti-codon loop contained conserved C^1^-U^2^-G^3^-U^4^-C^5^-A^6^ nucleotides followed by the presence of conserved A^1^-x-G^3^-U^4^ nucleotides in the variable region, G^1^-x-G^3^-x-G^5^ nucleotides in the Ψ-arm and U^1^-U^2^-C^3^-x-A^5^-x-C^7^ nucleotides in the Ψ-loop, respectively (Table 2). The C^7^ nucleotide in the Ψ-loop sometimes substituted with U^7^ nucleotide.

In tRNA^Glu^, the majorities of species were contained conserved G^1^ nucleotide in the acceptor arm (Table 2). In a few species, G^1^ nucleotide was substituted by A^1^ nucleotide at the first position. The U^8^ nucleotide in the acceptor arm was conserved whereas the 9th position was substituted by A^9^/G^9^ or C^9^ nucleotides (Table 3). The D-arm was devoid of any conserved nucleotide sequence whereas the D-loop contained conserved G^5^ nucleotide. The anti-codon loop contained conserved U^2^-U^3^-U^4^-C^5^ nucleotides, followed by the presence of conserved G^3^-x-G^5^ and U^1^-U^2^-C^3^-x-A^5^-x-U^7^ nucleotides in the Ψ-arm and Ψ-loop, respectively. The U^7^ nucleotide in the Ψ-loop sometimes substituted with the C^7^ nucleotide.

### Cyanobacterial tRNAs contain group I intron

**Figure 2:**
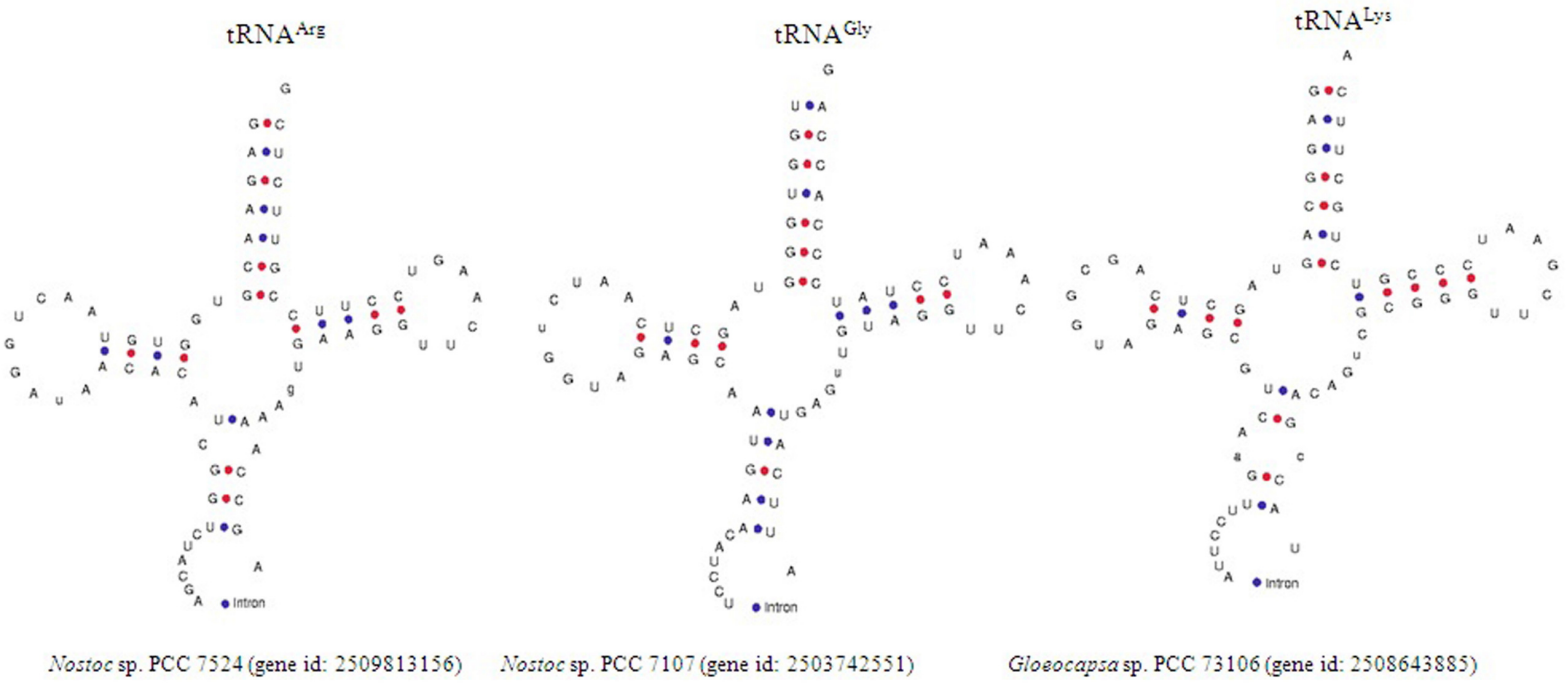
Figure representing the presence of group I intron in the cyanobacterial tRNA. *Nostoc* sp. PCC 7524 (gene id: 2509813156) was found to encode tRNA^Arg^, and *Nostoc* sp. PCC 7107 (gene id: 2503742551) was found to encode tRNA^Gly^ whereas, *Gleocapsa* sp. PCC 73106 (gene id: 2508643885) was found to encode for tRNA^Lys^. The red and blue marks indicate G-C and A-U bonding, respectively.

**Figure 3:**
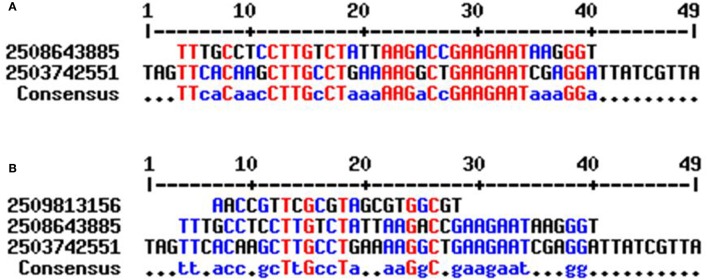
Sequence alignment of cyanobacterial group I intron. **(A)** Sequence alignment of cyanobacterial tRNA showed, the introns found in *Nostoc* sp. PCC 7107 (gene id: 2503742551) and *Gleocapsa* sp. PCC 73106 (gene id: 2508643885) share conserved consensus sequences **(B)** whereas the intron of *Nostoc* sp. PCC 7524 (gene id: 2509813156) did not share any conserved sequences with other introns. This showed that there are two different groups of cyanobacterial group I intron.

In our study, cyanobacterial tRNA was found to contain group I introns. The intron was present in the anti-codon loop region of the tRNA in the *Nostoc* sp. PCC 7524 (gene id: 2509813156), *Gloeocapsa* sp. PCC 73106 (gene id: 2508643885), and *Nostoc* sp. PCC 7107 (gene id: 2503742551) (Figure 2). tRNA^Arg^ (ACG) of *Nostoc* sp. PCC 7524 contained an intron from nucleotide 39–60 whereas in tRNA^Gly^ (TCC) of the *Nostoc* sp. PCC 7107 the intron was found from nucleotide 39–87. One of the genes encoding tRNA^Lys^ (CTT) of the *Gloeocapsa* sp. PCC 73106 was found to contain an intron, from nucleotide 38 to 74. Previous studies reported that an intron was present only in tRNA^Leu^ (UAA) and tRNA^fMet^ (UAC) of cyanobacterial tRNA (Paquin et al., 1997; Rudi and Jakobsen, 1999). However in our study, we found that the cyanobacterial group I intron was also present in tRNA^Arg^, tRNA^Gly^, and tRNA^Lys^ (Figure 2). Multiple sequence alignment has shown the presence of conserved T-x-G-x_2_-T and G-x-C motifs in the introns of cyanobacterial tRNA (Figure 3). Sequence alignment between the introns of *Gloeocapsa* sp. PCC 73106 (tRNA^Lys^) and *Nostoc* sp. PCC 7107 (tRNA^Gly^) showed the presence of conserved T-T-x_2_-C, C-T-T-G-x-C-T, A-A-G-x-C, G-A-A-G-A-A-T, and G-G motifs. The length of the intron ranged between 22 and 49 nucleotides. The introns of the *Nostoc* sp. PCC 7524 (gene id: 2509813156) and *Nostoc* sp. PCC 7107 (gene id: 2503742551) were found to be 22 and 38 nucleotides, respectively, whereas that of the *Gloeocapsa* sp. PCC 73106 (gene id: 2508643885) was 49 nucleotides long. No introns were found in other parts of the tRNA except for the anti-codon loop.

### The rate of transition of cyanobacterial tRNAs were higher than the rate of transversion

The genes of cyanobacterial tRNA are found to range from 55 to 96 nucleotides; a tRNA gene possessing only 55 nucleotides is the smallest gene reported thus far. Studying the substitution pattern of mutation is important for understanding the molecular basis of evolution. Therefore, we examined the substitutions (transition/transversion) of cyanobacterial tRNA. The mutation, which changes a purine (A<>G) nucleotide to another purine or a pyrimidine nucleotide to another pyrimidine (T<>C) is called transition, whereas a mutation that changes a purine nucleotide to a pyrimidine and vice versa, is called a transversion. In our study, we found that the rate of transition, in cyanobacterial tRNA, was higher than that of transversion (Table 4). The highest rate of transition was 42.05 in tRNA^Trp^ followed by 25.66 in tRNA^Cys^, where an adenine was substituted with a guanine (Table 4); the highest rate of transversion was 9.7 in tRNA^Ser^, where a uracil and a cytosine were substituted with a guanine (Table 4). The highest rate of transition from a guanine to an adenine was found in tRNA^Trp^ (28.89), whereas the highest rate of transition from a uracil to a cytosine was found in tRNA^Tyr^ (Table 4). The highest rate of transition from a cytosine to a uracil was found in tRNA^Tyr^. The lowest rate of transition was found in tRNA^Ser^ where a guanine was substituted with an adenine. Similarly, the lowest rate of transversion was found in tRNA^Trp^ where a uracil and a cytosine were substituted by an adenine (Table 4). The transition/transversion bias of tRNA^Trp^ was highest, followed by tRNA^Asp^ and tRNA^Tyr^ (Table 4).

**Table 4:** Maximum composite likelihood transition/transversion rate of cyanobacterial tRNAs.

	**A**	**U**	**C**	**G**
**GLYCINE**				
A	–	*4.61*	*4.76*	**17.77**
U	*3.43*	–	**17.54**	*5.25*
C	*3.43*	**16.99**	–	*5.25*
G	**11.59**	*4.61*	*4.76*	–
**SERINE**				
A	–	*6.6*	*7.18*	**9.66**
U	*6.19*	–	**12.92**	*9.7*
C	*6.19*	**11.88**	–	*9.7*
G	**6.17**	*6.6*	*7.18*	–
**THREONINE**				
A	–	*4.66*	*4.63*	**17.7**
U	*3.93*	–	**16.07**	*5.69*
C	*3.93*	**16.19**	–	*5.69*
G	**12.22**	*4.66*	*4.63*	–
**CYSTEINE**				
A	–	*3.74*	*5.37*	**25.66**
U	*4.2*	–	**10.05**	*5.46*
C	*4.2*	**7**	–	*5.46*
G	**19.73**	*3.74*	*5.37*	–
**TYROSINE**				
A	–	*2.19*	*2.65*	**17.47**
U	*1.96*	–	**28.31**	*3.1*
C	*1.96*	**23.4**	–	*3.1*
G	**11.04**	*2.19*	*2.65*	–
**ASPARAGINE**				
A	–	*4.73*	*4.39*	**19.84**
U	*3.73*	–	**14.51**	*6.1*
C	*3.73*	**15.63**	–	*6.1*
G	**12.12**	*4.73*	*4.39*	–
**GLUTAMINE**				
A	–	*4.47*	*5.18*	**10.59**
U	*3.72*	–	**23.69**	*5.97*
C	*3.72*	**20.46**	–	*5.97*
G	**6.59**	*4.47*	*5.18*	–
**ALANINE**				
A	–	*6.84*	*6.95*	**13.53**
U	*5.62*	–	**10.62**	*9.1*
C	*5.62*	**10.46**	–	*9.1*
G	**8.36**	*6.84*	*6.95*	–
**VALINE**				
A	–	*5.96*	*6.37*	**13.83**
U	*5.53*	–	**13.07**	*7.43*
C	*5.53*	**12.23**	–	*7.43*
G	**10.3**	*5.96*	*6.37*	–
**ASPARTATE**				
A	–	*2.11*	*2.66*	**20.62**
U	*1.64*	–	**27.74**	*3.27*
C	*1.64*	**21.99**	–	*3.27*
G	**10.33**	*2.11*	*2.66*	–
**LEUCINE**				
A	–	*5.68*	*5.92*	**15.54**
U	*5.04*	–	**13.02**	*7.86*
C	*5.04*	**12.5**	–	*7.86*
G	**9.96**	*5.68*	*5.92*	–
**ISOLEUCINE**				
A	–	*5.56*	*5.97*	**21.94**
U	*4.74*	–	**9.12**	*7.94*
C	*4.74*	**8.04**	–	*7.94*
G	**13.09**	*5.56*	*5.59*	–
**PROLINE**				
A	–	*3.61*	*4.38*	**22.18**
U	*2.82*	–	**18.54**	*5.95*
C	*2.82*	**15.27**	–	*5.95*
G	**10.49**	*3.61*	*4.38*	–
**PHENYLALANINE**				
A	–	*5.89*	*6.63*	**14.24**
U	*5.96*	–	**11.59**	*8.46*
C	*5.96*	**10.29**	–	*8.46*
G	**10.03**	*5.89*	*6.63*	–
**TRYPTOPHAN**				
A	–	*1.24*	*1.34*	**42.05**
U	*1.09*	–	**9.65**	*1.58*
C	*1.09*	**8.91**	–	*4.58*
G	**28.89**	*1.24*	*1.34*	–
**METHIONINE**				
A	–	*4.42*	*5.9*	**13.38**
U	*4.58*	–	**19.75**	*6.15*
C	*4.58*	**14.79**	–	*6.15*
G	**9.97**	*4.42*	*5.9*	–
**LYSINE**				
A	–	*4.77*	*5.02*	**16.76**
U	*3.97*	–	**16.71**	*5.34*
C	*3.97*	**15.88**	–	*5.34*
G	**12.46**	*4.77*	*5.02*	–
**ARGININE**				
A	–	*4.86*	*5.86*	**19.01**
U	*4.06*	–	**14.36**	*7.29*
C	*4.06*	**11.91**	–	*7.29*
G	**10.58**	*4.86*	*5.86*	–
**HISTIDINE**				
A	–	*5.66*	*5.89*	**20.01**
U	*3.86*	–	**12.2**	7.46
C	*3.86*	**11.71**	–	7.46
G	**10.35**	*5.66*	*5.89*	–
**GLUTAMATE**				
A	–	*4.97*	*6.81*	**19.43**
U	*4.45*	–	**12.61**	6.49
C	*4.45*	**9.19**	–	6.49
G	**13.32**	*4.97*	*6.81*	–
**ALL TRNAS**				
A	–	*5.65*	*6.28*	**16.97**
U	*4.91*	–	**12.2**	*7.64*
C	*4.91*	**10.98**	–	*7.64*
G	**10.9**	*5.65*	*6.28*	–

Analyses showed that the rate of transition of cyanobacterial tRNAs are higher than the rate of transversion. The values marked bold in the table represent the rate of “transition” whereas italicized are represents rate of “transversion.”

### Cyanobacteria species evolved via the loss of tRNA genes

**Figure 4:**
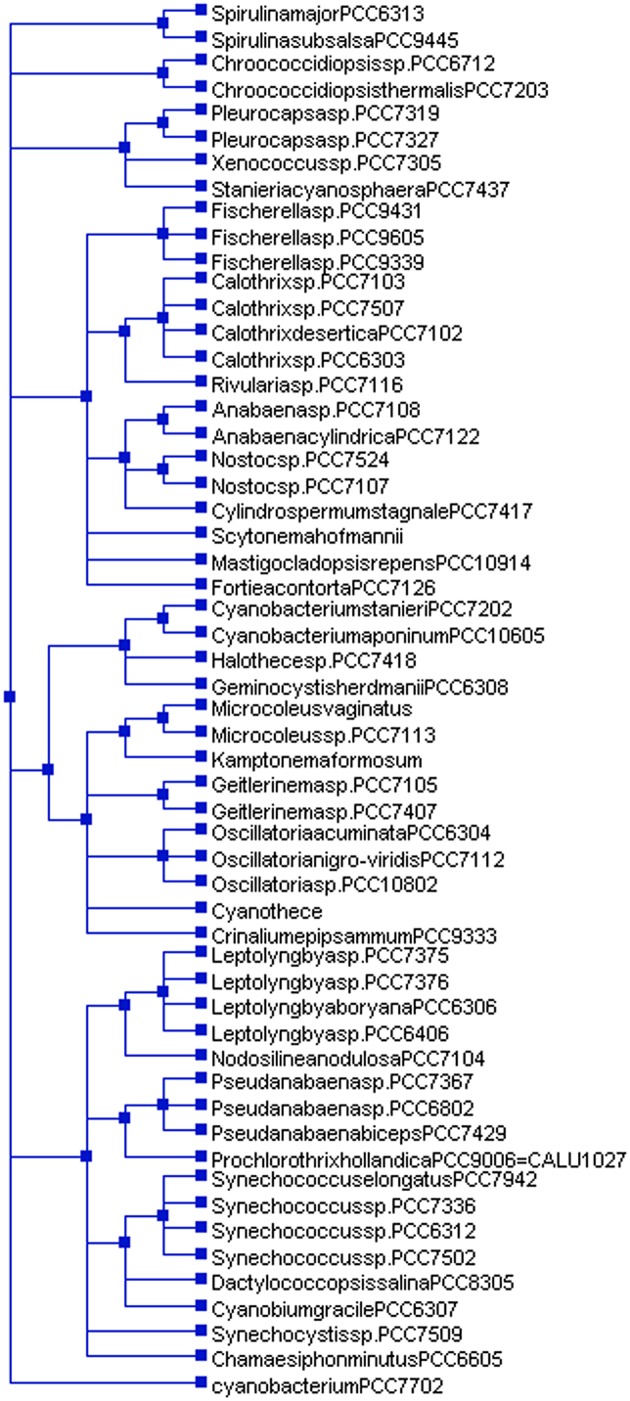
Species tree of cyanobacteria. The species tree was constructed using NCBI taxonomy browser (https://www.ncbi.nlm.nih.gov/Taxonomy/CommonTree/wwwcmt.cgi). *Cyanobacterium* sp. PCC 7702 in the species tree falls independently with regard to other cyanobacterial species. This show, cyanobacterial groups show polyphyletic origins.

In addition to substitution (transition/transversion), gene duplication and loss play crucial roles in the evolution of a gene. Duplication study of cyanobacterial tRNA showed that tRNA genes in all 20 tRNAs families, were duplicated by more than 50% (Table 5, Supplementary Figure 1). The highest duplication of 85% was observed in tRNA^Ile^, whereas the lowest duplication was observed in tRNA^His^ (Table 5). The highest percentage of conditional duplication was found in tRNA^Asn^ (20.75%), whereas the lowest percentage was found in tRNA^Ile^ (5.26%), followed by tRNA^Gln^ (5.68%) and tRNA^Asp^ (5.84%) (Table 5). Unlike duplication and conditional duplication, the highest percentage of losses with respect to the species tree (Figure 4), was found in tRNA^Asp^ (284.93%) followed by tRNA^Glu^ (280.76%). The low level of loss was found in tRNA^Arg^ (221.45%) followed by tRNA^Ala^ (226.63%).

**Table 5:** Duplication, conditional duplications and losses of cyanobacterial tRNAs.

**tRNA**	**k1 (Purines)**	**k2 (Pyrimidines)**	**R (Transition/Transversion Bias)**	**No. of sequences Studied**
Alanine	1.494	1.515	0.732	258
Arginine	2.607	2.449	1.207	255
Asparagine	3.389	3.268	1.618	90
Aspartate	6.971	10.501	4.131	84
Cysteine	4.699	1.87	1.65	78
Glutamate	3.951	2.82	1.626	87
Glutamine	1.626	4.461	1.499	94
Glycine	3.2	3.426	1.631	192
Histidine	2.832	2.25	1.199	68
Isoleucine	0.231	1.512	0.39	124
Leucine	1.845	2.114	0.964	343
Lysine	3.149	3.386	1.623	126
Methionine	1.805	3.28	1.231	202
Phenylalanine	1.619	1.661	0.811	89
Proline	3.708	4.158	1.825	204
Threonine	3.133	3.453	1.618	202
Tryptophan	26.77	7.30	8.409	83
Tyrosine	5.114	10.015	3.658	76
Serine	0.914	1.82	0.654	271
Valine	1.959	2.169	1.018	159

*Highest transition/transversion bias was found in tRNA^Trp^ whereas the lowest was found in tRNA^Ile^*.

### Cyanobacterial tRNAs may be polyphyletic

**Figure 5:**
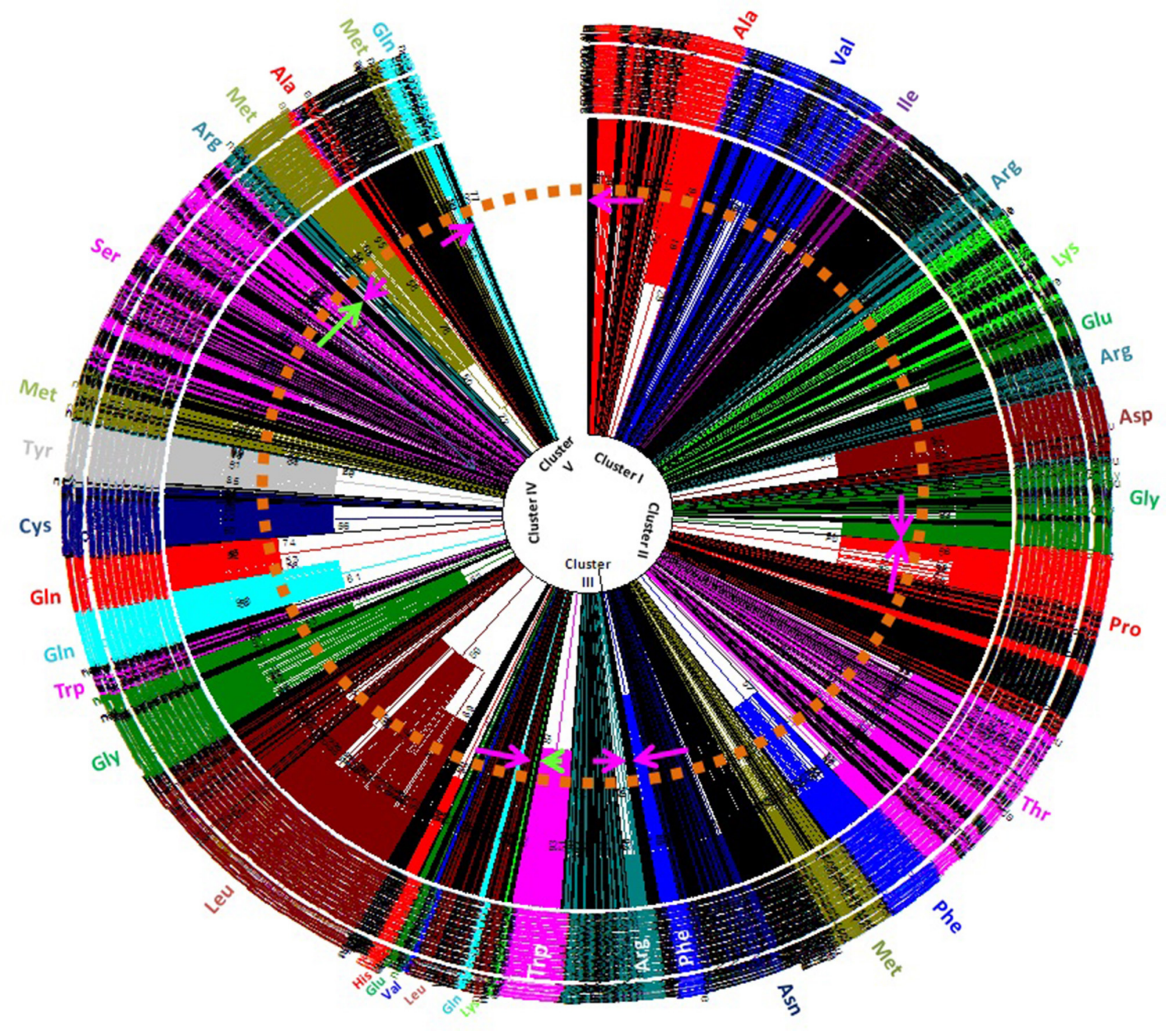
Phylogenetic tree of cyanobacterial tRNAs. Phylogenetic analysis revealed polyphyletic origin of cyanobacterial tRNA. Besides this, it also revealed that tRNA^Gln^, tRNA^Met^, tRNA^Ala^, and tRNA^Arg^ were most probably evolved earlier than others tRNAs. The other tRNAs mostly evolved from these tRNAs with subsequent modification (transition/transversion), duplication and eventual loss of old genes.

Understanding the evolution of cyanobacterial tRNA is important for delineating the evolution of tRNA, its evolutionary lineages, and subsequent diversification. In this study, we constructed a phylogenetic tree by examining all the tRNAs of the studied species (Figure 5). The phylogenetic tree was divided into five distinct clusters (Supplementary Figure 1). Cluster I contained tRNA^Ala^ (red), tRNA^Val^ (blue), tRNA^Ile^ (purple), tRNA^Arg^ (teal), tRNA^Lys^ (lime), tRNA^Glu^ (green), tRNA^Asp^ (maroon), and tRNA^Gly^ (green). Cluster II was found to contain tRNA^Pro^ (red), tRNA^Thr^ (fuchsia), tRNA^Phe^ (blue), tRNA^Met^ (olive), tRNA^Asn^ (navy), and tRNA^Phe^ (blue) (Supplementary Figure 1). Cluster III contained tRNA^Arg^ (teal) and tRNA^Trp^ (fuchsia) whereas cluster IV had tRNA^Lys^ (lime), tRNA^Leu^ (maroon), tRNA^Gln^ (aqua), tRNA^Leu^ (maroon), tRNA^Val^ (blue), tRNA^Glu^ (green), tRNA^His^ (red), tRNA^Ala^ (black), tRNA^Leu^ (maroon), tRNA^Gly^ (green), tRNA^Trp^ (fuschia), tRNA^Gln^ (aqua), tRNA^His^ (red), tRNA^Cys^ (navy), tRNA^Tyr^ (gray), tRNA^Met^ (olive), and tRNA^Ser^ (fuschia) (Supplementary Figure 1). Cluster V contained tRNA^Arg^ (teal), tRNA^Met^ (olive), tRNA^Ala^ (red), tRNA^Met^ (olive), and tRNA^Gln^ (aqua). In cluster I, tRNA^Ala^ and tRNA^Val^ were present together with tRNA^Val^ and tRNA^Ile^. In cluster I tRNA^Arg^ was shared by tRNA^Asp^, and tRNA^Gly^. Cluster II was shared by tRNA^Pro^, tRNA^Thr^, tRNA^Phe^, tRNA^Met^, and tRNA^Asn^ (Supplementary Figure 1). Cluster III was shared by tRNA^Arg^ and tRNA^Trp^. Cluster IV contained tRNA^Lys^, tRNA^Leu^, tRNA^Gln^, tRNA^Val^, tRNA^His^, tRNA^Ala^, tRNA^Gly^, tRNA^Trp^, tRNA^Gln^, tRNA^Cys^, tRNA^Tyr^, tRNA^Met^, and tRNA^Ser^. Cluster V contained tRNA^Ala^, tRNA^Met^, tRNA^Gln^, and tRNA^Arg^. tRNA^Ala^ was distributed in clusters I, V, and VI whereas tRNA^Gln^ was distributed in clusters IV, V, and VI. tRNA^Val^, tRNA^Lys^, and tRNA^Gly^ were distributed in clusters I and IV; tRNA^Asn^ was distributed in cluster II and IV, and tRNA^Trp^ was distributed in cluster III and IV (Supplementary Figure 1). Due to low bootstrap values in few clades, it was difficult to infer the phylogenetic result properly. Therefore, we collapsed the clades with low bootstrap values (cut-off values for condensed tree was 50%) (Figure 5, Supplementary Figure 1). The overall tree architecture remained unchanged post-collapsing the clades with 50% cut-off values. Several potentially novel evolutionary trends were found in the phylogenetic analysis of cyanobacterial tRNA. In between tRNA^Met^ and tRNA^Asn^ of cluster II, several other tRNAs were also found. In cluster V, tRNA^Asp^ and tRNA^Asn^ were found in close to tRNA^Met^, suggesting their possible evolution from tRNA^Met^. Similarly, a few tRNAs^Val^ (*Microcoleus* sp. PCC 7113, gene id: 2509433894; *Leptolyngbya boryana* PCC 6306, gene id: 2509801628) were present close to tRNA^Ala^. This suggests that, tRNA^Val^ has likely evolved from tRNA^Ala^. In cluster V, tRNA^Gly^ (*Microcoleus* sp. PCC 7113, gene id: 2509433891) was clustered with tRNA^Met^, reflecting it's evolution from tRNA^Met^.

## Discussion

Nucleotide sequence conservation is an important phenomenon that demonstrates conserved functional role. Therefore, understanding the conserved nucleotide consensus sequences in cyanobacterial tRNA was very important. In this study we found that except for tRNA^Asn^, tRNA^Leu^, tRNA^Gln^, and tRNA^Met^, all the tRNAs were contained conserved G^1^ nucleotide at the 1st position in the acceptor arm. tRNA^Leu^ and tRNA^Met^ did not contain any conserved nucleotides at the 1st position whereas tRNA^Asn^ and tRNA^Gln^ contained a conserved U^1^ nucleotide at the 1st position. With a few exceptions, the G^2^ nucleotide was conserved at the 2nd position in tRNA^Ala^, tRNA^Val^, tRNA^Ile^, tRNA^Phe^, tRNA^Tyr^, tRNA^Ser^, tRNA^Gln^, tRNA^Lys^, tRNA^His^, tRNA^Arg^, and tRNA^Asp^. The nucleotide C^2^ was conserved in tRNA^Gly^ and tRNA^Thr^ whereas tRNA^His^ contained either a C^2^ or a U^2^ nucleotide. At the 3rd position, the G^3^ nucleotide was conserved in tRNA^Gly^, tRNA^Ala^, tRNA^Pro^, tRNA^Ile^, tRNA^Tyr^, tRNA^Lys^, and tRNA^Asp^, whereas tRNA^Phe^ contained a C^3^; tRNA^His^ contained C^3^/U^3^; whereas tRNA^Ser^ contained an A^3^ nucleotide. At the 4th position of the acceptor arm, tRNA^Val^ and tRNA^Ile^ contained the conserved C^4^ nucleotide whereas tRNA^Tyr^ contained a U^4^ nucleotide and tRNA^Pro^ contained the G^4^ nucleotide. In tRNA, the C^5^ nucleotide was conserved, whereas tRNA^Phe^ and tRNA^Gln^ contained the conserved G^6^ nucleotide. In all canonical tRNA, 1st, 2nd, and 3rd base of the acceptor arm pairs with the 72nd, 71st, and 70th base, respectively of the acceptor arm. Their conserved structure is highly important to have proper functional clover leaf-like structure of tRNA. At the 7th position, tRNA^Pro^ and tRNA^His^ contained conserved G^7^ nucleotide. In tRNA^Tyr^, all the nucleotides in the positions 1–5 were conserved across the species. In the 1st and 2nd positions of the acceptor arm of tRNA, the G^1^-G^2^ conserved consensus sequence present in tRNA^Ala^, tRNA^Val^, tRNA^Ile^, tRNA^Tyr^, tRNA^Ser^, tRNA^Lys^, tRNA^Arg^, and tRNA^Asp^ whereas the G^1^-C^2^ conserved consensus sequence present in tRNA^Gly^, tRNA^Phe^, tRNA^Thr^, and tRNA^Lys^. The nucleotide, at the 2nd position of the acceptor arm, was substituted with either G^2^ or C^2^, whereas only tRNA^His^ had a U^2^ at the 2nd position. Only tRNA^Asn^ and tRNA^Gln^ contained a U^1^ nucleotide at the 1st position of the acceptor arm whereas tRNA^Tyr^ contained conserved U^4^ nucleotide at the 4th position and tRNA^His^ contained the conserved U^2^ nucleotide at the 2nd position (Table 2). Except the above-mentioned four tRNAs, none of the other tRNAs were found to encode U nucleotide in the acceptor arm. Previously, the 8th position of tRNA between the acceptor arm and the D-arm was thought to contain a conserved U^8^ nucleotide (Table 3). In this study, the majority of tRNAs were found to contain a conserved U^8^ nucleotide. However, tRNA^His^ was found to contain either a U^8^ or an A^8^ whereas tRNA^Glu^ contained a U^8^, C^8^ or a G^8^ nucleotide (Table 3). This showed that the 8th position of cyanobacterial tRNA can be substituted with any nucleotide. Similarly, at the 9th position, the majority of tRNAs were found to contain a conserved A^9^ nucleotide (Table 3). However, tRNA^Leu^, tRNA^Tyr^ and tRNA^Ser^ were found to contain a G^9^ nucleotide whereas tRNA^Thr^, tRNA^Lys^, and tRNA^Arg^ contained an A^9^ or a G^9^ nucleotide (Table 3). tRNA^His^ contained an A^9^ or a C^9^ nucleotide at the 9th position whereas tRNA^Cys^ and tRNA^Gln^ contained either a A^9^, C^9^, or a G^9^ (Table 3). tRNA^Met^ contained either an A^9^, G^9^ or a U^9^ nucleotide whereas tRNA^Glu^ contained an A^9^, C^9^, or a U^9^ nucleotide at the 9th position. This negated the previous assumption about the conservation of the U^8^ nucleotide in cyanobacterial tRNA and showed that it can be prone to variations; the major variation were observed in tRNA^Glu^ which was found to contain varied nucleotides at 8th and 9th position.

In the D-arm, the majority of tRNA contained a conserved G^1^ nucleotide at the 1st position except for tRNA^Gly^, tRNA^Pro^, tRNA^Val^, tRNA^Met^, tRNA^Gln^, tRNA^Lys^, and tRNA^Glu^. In total of 20 tRNA families, 10 families were found to contain a conserved C^2^ nucleotide at the 2nd position (Table 2). These were tRNA^Ala^, tRNA^Val^, tRNA^Ile^, tRNA^Phe^, tRNA^Ser^, tRNA^Thr^, tRNA^Cys^, tRNA^Asn^, tRNA^Lys^, and tRNA^His^ whereas tRNA^Met^ was found to contain a G^2^ and tRNA^Trp^ contained a U^2^ nucleotide at the 2nd position (Table 2). With respect to the first two nucleotides in the D-arm, the G^1^-C^2^ conserved consensus sequence can be found in tRNA^Ala^, tRNA^Ile^, tRNA^Phe^, tRNA^Ser^, tRNA^Thr^, tRNA^Cys^, tRNA^Asn^, and tRNA^His^. At the 3rd position, the U^3^ nucleotide conserved in tRNA^Ala^, tRNA^Ile^, tRNA^Phe^, tRNA^Asn^, and tRNA^Asp^. At the 4th position of the D-arm, the C^4^ nucleotide was found to be conserved in tRNA^Ala^, tRNA^Val^, tRNA^Ile^, tRNA^Phe^, tRNA^Tyr^, tRNA^Thr^, and tRNA^Asp^ (Table 2). The D-stem expected to have rigid structure due to high G/C content, but due to the presence of more A-U and G-U base pairing, it seems rather weak (Hardt et al., 1993). Previous study reported that D-arm play important role in recognition of aminoacyl-tRNA synthetase and is highly variable and possess unusual conformation due to over-crowding of G residue (Smith and Yarus, 1989; Hardt et al., 1993). However, in our study we found that D-arm is also highly conserved, suggesting its common functional role.

Except for tRNA^Met^ and tRNA^Glu^, 18 tRNA families were found to contain a conserved A^1^ nucleotide at the 1st position in the D-loop. At the 2nd position, the G^2^ nucleotide was conserved in tRNA^Pro^, tRNA^Val^, tRNA^Phe^, and tRNA^Tyr^, whereas tRNA^Thr^ contained a U^2^ nucleotide at the 2nd position (Table 2). In the 3rd position, the U^3^ nucleotide was conserved in tRNA^Phe^, and tRNA^Tyr^ and the G^3^ nucleotide was conserved in tRNA^Cys^, tRNA^Asn^, and tRNA^His^. In the 4th position of the D-loop, the G^4^ nucleotide was conserved in tRNA^Gly^, tRNA^Tyr^, tRNA^Thr^, tRNA^Asn^, tRNA^Gln^, tRNA^Lys^, tRNA^Arg^, and tRNA^Asp^ whereas at the same position tRNA^Phe^ contained conserved U^4^ nucleotide (Table 2). At the 5th position, the G^5^ nucleotide was found to be conserved in tRNA^Gly^, tRNA^Ala^, tRNA^Leu^, tRNA^Phe^, tRNA^Tyr^, tRNA^Trp^, tRNA^Ser^, tRNA^Cys^, tRNA^Lys^, tRNA^His^, tRNA^Asp^ and tRNA^Glu^. The majority of tRNAs were found to contain a conserved G^5^ nucleotide whereas tRNA^Thr^ contain a U^5^ nucleotide. At the 6th position, tRNA^Ala^, tRNA^Val^, tRNA^Leu^, tRNA^Ile^, tRNA^Met^, tRNA^Phe^, tRNA^Ser^, tRNA^Cys^, and tRNA^His^ contained the G^6^ nucleotide whereas tRNA^Tyr^, and tRNA^Asp^ contained the U^6^ nucleotide. At the 7th position of the D-loop, the U^7^ nucleotide was found to be conserved in tRNA^Phe^, tRNA^Tyr^, tRNA^Cys^, and tRNA^His^ whereas the 8th position had a conserved A^8^ nucleotide in tRNA^Ala^, tRNA^Leu^, tRNA^Tyr^, tRNA^Arg^ and tRNA^Asp^. The A^9^ nucleotide was conserved in tRNA^Val^, and tRNA^Ile^. This indicates that a great variation was present in the nucleotide composition of the D-loop. However, the A^1^ nucleotide was conserved in the majority of the cases, whereas conservation of nucleotides in the other parts of the D-loop was specific to an individual tRNA family. The D-loop interacts with the Ψ-loop via long range interactions where G^18^ and G^19^ interact with Ψ^55^ and C^56^, respectively (Hanawa-Suetsugu et al., 2001). Although the position is dynamic, except for tRNA^Gln^, all tRNAs were contained at least one conserved G nucleotide in the D-loop, suggesting their common functional role.

The anti-codon arm of tRNA was less conserved than other parts. In tRNA^Phe^, tRNA^Trp^ and tRNA^His^, the G^4^ nucleotide was found to be conserved, whereas tRNA^Trp^ had a conserved U^5^ nucleotide and in tRNA^His^, the G^5^ nucleotide was conserved. The anti-codon loop, which reads the codon of an mRNA during protein translation, is the most important part of tRNA. The C^1^ nucleotide was found to be conserved at the 1st position of the anti-codon loop in tRNA^Ile^, tRNA^Met^, tRNA^Trp^, tRNA^Cys^, tRNA^Asn^, tRNA^Lys^, and tRNA^Asp^. Additionally, the U^1^ nucleotide at the 1st position of the anti-codon loop, was found to be conserved in tRNA^Pro^, tRNA^Val^, tRNA^Gln^, tRNA^His^, and tRNA^Arg^ (Table 2). Occasionally, tRNA^Arg^ contained a C^1^ nucleotide in the anti-codon loop. All the tRNAs were found to contain a conserved U^2^ nucleotide at the 2nd position of the anti-codon loop. The A^6^ nucleotide, at the 6th position of the anti-codon loop, was conserved in tRNA^Gly^, tRNA^Ala^, tRNA^Val^, tRNA^Ile^, tRNA^Met^, tRNA^Trp^, tRNA^Ser^, tRNA^Thr^, tRNA^Cys^, tRNA^Asn^, tRNA^Lys^, and tRNA^Asp^ (Table 2). In the 7th position of the anti-codon loop, the A^7^ nucleotide was conserved in tRNA^Ile^, tRNA^Met^, tRNA^Trp^, tRNA^Thr^, tRNA^Cys^, tRNA^Asn^, and tRNA^Lys^. The presence of a conserved A^6^ nucleotide at the 6th position suggests the possible entry site for group I intron in the anti-codon loop.

Previous study reported that the variable loop of tRNA is dynamic in nature and does not contain any conserved nucleotides. However, in our study we found that the variable loop contained conserved nucleotides at the specific positions at the level of individual tRNA family (Table 2). tRNA^Val^ and tRNA^Phe^ were found to contain the conserved G^3^-U^4^-C^5^ nucleotides at the 3rd, 4th, and 5th positions of the variable loop, whereas tRNA^Pro^ contained the conserved G^3^-x-C^5^ nucleotides at 3rd and 5th position (Table 2). In tRNA^Ile^ and tRNA^Asn^, the G^3^-U^4^ nucleotides were found to be conserved at 3rd and 4th positions. At the 5th position, the C^5^ nucleotide was conserved in tRNA^Pro^, tRNA^Val^, tRNA^Phe^, and tRNA^Cys^ (Table 2). This study indicates that the G^3^, U^4^, and C^5^ nucleotides at 3rd, 4th, and 5th position, respectively, were the most likely to be conserved in the variable loop of the cyanobacterial tRNA.

All the tRNAs were found to contain a conserved G^5^ nucleotide in the Ψ-arm. Several tRNAs contained a conserved G^4^ nucleotide at the 4th position whereas others was found to contain conserved G^3^ nucleotide. The conserved G nucleotide at the 4th and 5th position was detected as the G^4^-G^5^ configuration in tRNA^Pro^, tRNA^Val^, tRNA^Ile^, tRNA^Tyr^, tRNA^Ser^, tRNA^Gln^, tRNA^Lys^, tRNA^His^, and tRNA^Arg^.

The Ψ-loop of all the tRNA contained the conserved U^1^-U^2^-C^3^-x-A^5^ nucleotides at the 1st, 2nd, 3rd, and 5th positions, respectively. Few other tRNAs were found to contain the extended conserved U^1^-U^2^-C^3^-x-A^5^-x-U^7^ sequence from the 1st to the 7th position of the Ψ-loop (Table 2). The tRNA containing the conserved U^1^-U^2^-C^3^-x-A^5^-x-U^7^ nucleotides were tRNA^Leu^, tRNA^Ile^, tRNA^Met^, tRNA^Tyr^, tRNA^Trp^, tRNA^Ser^, tRNA^Cys^, tRNA^Asn^, tRNA^Lys^, tRNA^His^, tRNA^Arg^, and tRNA^Glu^ (Table 2). tRNA^Pro^ was found to contain the conserved U^1^-U^2^-C^3^-x-A^5^-A^6^-U^7^ consensus sequence, where the A^6^ nucleotide was found to be conserved at the 6th position. In tRNA^Asp^, it was found to contain conserved U^1^-U^2^-C^3^-x-A^5^-x-C^7^ consensus sequence in which the U^7^ nucleotide substituted with a C^7^ nucleotide (Table 2). These analyses show that tRNAs were conserved in the acceptor arm, D-arm, D-loop, anti-codon arm, anti-codon loop, the variable loop and the Ψ-arm at the individual family level with majority of tRNAs showing greater conservation in the Ψ-loop.

In spite of the presence of highly conserved genetic architecture of cyaobacterial tRNA, they also found to possess introns within it. Previous studies reported that an intron was present only in tRNA^Leu^ (UAA) and tRNA^fMet^ (UAC) of cyanobacterial tRNA (Paquin et al., 1997; Rudi and Jakobsen, 1999). However in our study, we found that the cyanobacterial group I intron was also present in tRNA^Arg^, tRNA^Gly^, and tRNA^Lys^ (Figure 3). Because cyanobacteria are a prokaryotic organism, the intron, present in cyanobacterial tRNA can be compared with the group I intron in the plastid. Previous studies reported that the group I intron, found in the plastid, are immobile (Rudi and Jakobsen, 1999). The group I introns found in various other locations including the mitochondria, nucleus, bacteria, and bacteriophages share conserved sequence motifs and have evolved via lateral gene transfer (Rudi and Jakobsen, 1999). Cyanobacterial group I introns were also possessed conserved motifs. Rudi and Jakobsen (1999) have classified the group I intron as intron element P, Q, R, and S. Cluster analysis revealed that tRNA^Arg^ of the *Nostoc* sp. PCC 7524 were grouped in intron element P, whereas the introns of the *Nostoc* sp. PCC 7107 and *Gloeocapsa* sp. PCC 73106 were clustered independently. None of these were clustered with intron elements Q, R, or S. This suggests that there may be another conserved intron element in the cyanobacteria which is yet to be elucidated. The presence of an intron, in the anti-codon loop of cyanobacterial tRNA agrees with the exon theory of the evolution of tRNA genes where modern tRNA is reported to have evolved by duplication of one half of tRNA (Di Giulio, 1992). Report suggests that the introns of cyanobacterial tRNA moved via lateral gene transfer (Marck and Grosjean, 2002).

Self-splicing group I intron of cyanobacterial tRNA^fMet^ in genera *Dermocarpa, Scytonema*, and *Synechocystis* possess open reading frame (ORF). The intron of *Synechocystis* possesses ORF of 150 codons. The introns in cyanobacteria distributed sporadically without any correlation between relatedness in intron sequence corroborates with the lateral transfer of intron (Dujon, 1989; Biniszkiewicz et al., 1994). Sporadic occurrence of these group I introns in homologous genes of closely related species show consistent similarity in the mobility. The group I introns are suggested to be either mobile and ancient in origin (Rudi and Jakobsen, 1999). Closely related group I intron are inserted in the UAA anit-codon of tRNA^Leu^ of cyanobacteria and chloroplast (Biniszkiewicz et al., 1994). Study of introns from 22 cyanobacterial strains by Rudi and Jakobsen (1999) demonstrated relatively recent gain and/or loss of intron in some cyanobacterial lineage (Rudi and Jakobsen, 1999). The presence of self-splicing group I intron in tRNA^Leu^ at the same position of the same gene in cyanobacteria and chloroplast indicated that this intron predates the invasion of eukaryotic cells by endosymbiosis (Reinhold-Hurek and Shub, 1992). The group I intron of tRNA^Leu^ have higher homology with the group I intron of tRNA^Ile^ (CAU) and tRNA^Arg^ (CCU) (Rudi and Jakobsen, 1997, 1999).

Evolution of a gene occurred through random mutation/substitution. The pattern and frequencies of nucleotide substitution is largely depends on the mutational events of the gene (Shimizu et al., 1989; Zhang and Gerstein, 2003; Arnheim and Calabrese, 2009). Although transitions are more frequent in coding genes because they are less likely to result in amino acid substitutions due to a wobble in the genetic code, the presence of a higher rate of transition in cyanobacterial tRNA is intriguing. A universal bias may favor transition over transversion and a diverse rate of transitions/transversions, combined with the bias may help to explain the multiple evolutionary rates and lineages. The rates of transition/transversion bias of tRNA^Trp^, tRNA^Asp^, and tRNA^Tyr^ were 8.409, 4.131, and 3.658, respectively; these were quite higher than the rates of other tRNAs (Table 6). At a low level of genetic divergence, the transition/transversions bias remains high, whereas at a high level of genetic divergence, the transition/transversion bias remains low (Yang and Yoder, 1999). This indicates that tRNA^Trp^, tRNA^Asp^, and tRNA^Tyr^ have a very low level of genetic divergence, whereas other tRNAs have a very high rate of genetic divergence. Therefore, cyanobacterial species have only a fewer number of tRNA^Trp^, tRNA^Asp^, and tRNA^Tyr^ genes in their genome compared to other tRNAs and rest of the tRNAs have a very high rate of divergence. Due to high genetic divergence, the frequency of other tRNAs are more abundant than tRNA^Trp^, tRNA^Asp^, and tRNA^Tyr^. Silent mutation can have significant impact on exhibiting genetic divergence which accompany through selection/genetic drift or novel adaptation (Palumbi, 1994).

**Table 6:** Transition/transversion bias of cyanobacterial tRNAs.

**tRNA**	**D/L score**	**Duplications**	**Conditional duplications**	**Losses**
Alanine	797.5	163 (66.8%)	15 (6.14%)	553 (226.63%)
Arginine	712.5	131 (56.22%)	18 (7.72%)	516 (221.45%)
Asparagine	260.0	44 (53.01%)	11 (20.75%)	194 (233.73%)
Aspartate	287.5	53 (67.94%)	5 (6.84%)	208 (284.93%)
Cysteine	229.5	41 (59.42%)	4 (5.79%)	168 (243.47%)
Glutamate	304.5	57 (73.07%)	9 (11.53%)	219 (280.76%)
Glutamine	292.0	50 (56.81%)	5 (5.68%)	217 (246.59%)
Glycine	641.5	115 (65.34%)	17 (9.65%)	469 (266.47%)
Histidine	197.0	32 (52.45%)	7 (11.47%)	149 (244.26%)
Isoleucine	453.6	97 (85.08%)	6 (5.26%)	308 (270.17%)
Leucine	1081.5	193 (61.66%)	25 (7.98%)	792 (253.03%)
Lysine	372.5	67 (56.77%)	9 (7.62%)	272 (230.50%)
Methionine	598.0	108 (59.34%)	19 (10.43%)	436 (239.56%)
Phenylalanine	276.5	47 (56.62%)	8 (9.63%)	206 (248.19%)
Proline	699.5	133 (70.74%)	16 (8.51%)	500 (265.95%)
Serine	788.5	139 (55.60%)	23 (9.2%)	580 (232.00%)
Threonine	596.5	107 (57.83%)	15 (8.10%)	436 (235.67%)
Tryptophan	284.0	54 (70.12%)	5 (6.49%)	203 (263.63%)
Tyrosine	212.0	38 (57.57%)	6 (9.09%)	155 (234.84%)
Valine	485.5	85 (57.82%)	19 (12.92%)	358 (243.53%)

*Result showed loss of cyanobacterial tRNA gene predominate the duplication and conditional duplication event*.

Understanding the process of evolution and function of new gene is important in genomics and evolutionary biology; gene duplication is a powerful process in generating novel gene function via sub-functionalization and neo-functionalization, whereas loss of a gene can greatly shape the gene family (Lynch and Conery, 2000; Niimura and Nei, 2007; Rasmussen and Kellis, 2012; Teufel et al., 2016). Gene duplication is a fundamental process in the evolution of novel species, especially in eukaryotes in which it plays major roles in creating novel gene functions (Cotton and Page, 2005; Blomme et al., 2006; Zhao et al., 2015). Similarly, the loss of a gene occurs via segmental deletion, and pseudogenization preserves a minimum number of functional gene copies (Ohno, 1970; Lynch and Conery, 2000; Ting et al., 2004; Cotton and Page, 2005; Blomme et al., 2006; Demuth et al., 2006). Due to the lowest percentage of duplication, the genome of the cyanobacteria encodes only one to two tRNA^His^ genes per genome, whereas the number of tRNA^Ile^ genes is more numerous due to highest percentage of duplication (Table 5). The low level of duplication and conditional duplication may responsible for the low frequency in the occurrence of tRNA^His^, tRNA^Asn^, and tRNA^Asp^ in the cyanobacterial genome. Coupled with the low percentage of duplication, conditional duplication and the high percentage of gene loss led to the low frequency of tRNA^His^, tRNA^Asn^, tRNA^Gln^, and tRNA^Asp^ genes in the cyanobacterial genome. Similarly, low percentage of gene loss, coupled with the high percentage of gene duplication and conditional duplication in tRNA^Arg^ and tRNA^Ala^, resulted multiple and variable copies of tRNA^Arg^ and tRNA^Ala^ in the cyanobacterial genome (Table 5). Previous studies showed that tRNAs are the classic example of gene families that were continuously duplicated and lost (Withers et al., 2006; Tang et al., 2009; Bermudez-Santana et al., 2010; Rogers et al., 2010). The operon or tRNA clusters are highly dynamic and unstable genomic region. Therefore, it became an easy target for duplication or loss event. However, in cyanobacteria, the loss event was predominant over the duplication and conditional duplication.

Numerous studies have been conducted on the evolution of tRNA. Reports suggest that tRNA hairpin structures were the precursors of tRNA, having evolved from the combination of different sub-structures, where the top half (acceptor arm) of the tRNA is considered older than the bottom half containing the anti-codon arm (Sun and Caetano-Anollés, 2008). According to the “genomic tag” hypothesis, the upper half of the tRNA harbors the ancestral genomic information and the bottom half provides the specificity for the genetic code (Weiner and Maizels, 1987). The hairpin structure having the anti-codon loop may have evolved as an intermediary in the process of protein synthesis. Introns in tRNA are usually present in the anti-codon loop, and the 5′ and 3′ halves of the tRNA were likely the mini-genes before their merger (Di Giulio, 1992, 2004). The presence of an intron in tRNA suggests that the two modules of tRNA were separated by introns; hence, tRNA is the exon part of the gene described by the “exonic gene theory” of the origin of tRNA (Di Giulio, 1995). The tRNA molecule is universally present in all cellular organisms and is transferred by “horizontal gene transfer.” It's ubiquitous presence in all cellular lineages strongly supports its early origin, explaining its presence in organisms of early origin. Fossil record suggests that cyanobacteria are older than 2 billion years and indirect evidence suggests that they may be older than 2.45 billion years (Amard and Bertrand-Sarfati, 1997; Bekker et al., 2004; Schirrmeister et al., 2011; Mohanta et al., 2017). The presence of tRNA^Arg^ and tRNA^Met^ in clusters I, III, V, and VI in the phylogenetic tree reflected their ancient origin (Figure 5); hence, their co-distribution in different clusters may have been followed by subsequent evolution of tRNA^Gln^ and tRNA^Ala^. The presence of tRNA^Arg^ and tRNA^Met^ in multiple clusters vividly illustrates that they have undergone duplication and subsequently diverged into different species. The distribution of tRNA^Ile^, tRNA^Glu^, tRNA^Asp^, tRNA^pro^, tRNA^Thr^, tRNA^Gly^, tRNA^Trp^, tRNA^His^, tRNA^Cys^, tRNA^Tyr^, and tRNA^Ser^ was confined to a single cluster only (Figure 5). This suggests that they have evolved recently, compared with tRNA^Met^, tRNA^Arg^, tRNA^Ala^, and tRNA^Gln^ (Figure 5). In cluster I, tRNA^Arg^ grouped twice and present close to tRNA^Asp^ and tRNA^Gly^, suggesting their evolution from tRNA^Arg^ (Figure 5). In cluster I, tRNA^Ala^ was present close to tRNA^Val^; this suggests that tRNA^Val^ evolved from tRNA^Ala^. In cluster II, tRNA^pro^ was grouped separately whereas tRNA^Thr^, tRNA^Phe^, and tRNA^Asn^ were grouped with tRNA^Met^, suggesting their evolution from tRNA^Ala^ (Figure 5). Cluster III contained tRNA^Arg^ and tRNA^Trp^, suggesting possible evolution of tRNA^Trp^ from tRNA^Arg^. In cluster IV, tRNA^Gln^ present close to tRNA^His^, and tRNA^Met^ present closer to tRNA^Tyr^ and tRNA^Cys^. This suggests that tRNA^His^ may have evolved from tRNA^Gln^ whereas tRNA^Tyr^ and tRNA^Cys^ may have evolved from tRNA^Met^. tRNA^Ser^ found in cluster IV shared a branch with tRNA^Met^, suggesting the evolution of tRNA^Ser^ from tRNA^Met^. tRNA^Gln^, tRNA^Met^, and tRNA^Ala^ were found together in cluster V, suggesting that these tRNAs may have coevolved together, and later diversified and evolved into other tRNAs. These tRNAs were grouped with several pseudo-tRNAs in the cluster of tRNA^Asn^ (Figure 5, Supplementary Figure 1). This suggests that these tRNAs are likely in the verge of becoming pseudo-tRNAs and will be lost from the evolutionary lineage. tRNA^Phe^ was present as sister branch to tRNA^Asn^ in cluster III, suggesting their evolution from tRNA^Asn^. Several tRNAs^Lys^, tRNAs^His^, and tRNAs^Ala^ in cluster IV were grouped with the pseudo-tRNAs, suggesting their possible loss of function and, hence, extinction from the evolutionary lineage by pseudogenization. However, the evolutionary history of cyanobacterial tRNAs may be more complicated. The presence of common secondary and tertiary structures occurring with an invariant number of nucleotide residues, 20 invariant gene families, and similar functional roles during the translation is the phenomena that are complicated to delineate. It is assumed that for a total of 20 tRNA gene families, there would be 20 branches, corresponding to a group in each tRNA gene family, in the phylogenetic tree. However, this assumption is incorrect. The differences between tRNA families, with regard to their respective amino acid and tRNA specificity were found overlap significantly. The non-isoacceptor tRNAs shared the phylogenetic groups with the isoacceptors, suggesting their evolution from multiple lineages. This shows that tRNAs had evolved by multiple duplication with extensive genetic mutation and subsequent loss of old genes.

## Conclusion

Sequence analysis revealed that cyanobacterial tRNAs were dynamic and transiently conserved within their respective tRNA families. However, the U^1^-U^2^-C^3^ conserved consensus sequence in the Ψ-loop was found in all of the studied tRNAs. Several cyanobacterial tRNAs contained a group I intron in the anti-codon region; further study is required to discover more introns and to understand their specific genomic and evolutionary aspects. The rate of mutational transition of cyanobacterial tRNAs was higher than that of transversion; cyanobacterial tRNA may have evolved polyphyletically by loss of tRNA genes.

## Author contributions

TM: Conceived the idea, performed the study, analyzed data, drafted and revised the manuscript. AS: Revised the manuscript. FA: Revised the manuscript. HB: Revised the manuscript.

### Conflict of interest statement

The authors declare that the research was conducted in the absence of any commercial or financial relationships that could be construed as a potential conflict of interest.

## Supplementary material

Supplementary Figure 1 Phylogenetic tree of cyanobacterial tRNAs. The phylogenetic clades with low bootstrap replicates were collapsed with 50% cutoff values.

Supplementary data 1 Supplementary data showing genomic tRNA sequences of 61 cyanobacterial species.
