# Corrigendum: Novel Genomic and Evolutionary Perspective of Cyanobacterial tRNAs

**DOI:** 10.3389/fgene.2020.00202

**Published:** 2020-03-10

**Authors:** Tapan K. Mohanta, Asad S. Syed, Fuad Ameen, Hanhong Bae

**Affiliations:** ^1^School of Biotechnology, Yeungnam University, Gyeongsan, South Korea; ^2^Department of Botany and Microbiology, College of Science, King Saud University, Riyadh, Saudi Arabia

**Keywords:** cyanobacteria, tRNA, evolution, intron, transition, transversion

In the original article, the Tables 5, 6 were inadvertently given the wrong legends. The legend for Table 5 is for Table 6 and vice versa. The tables with their corrected legends appear below.

**Table 5:** Duplication, conditional duplication, and losses of cyanobacterial tRNAs.

**tRNA**	**D/L score**	**Duplications**	**Conditional duplications**	**Losses**
Alanine	797.5	163 (66.8%)	15 (6.14%)	553 (226.63%)
Arginine	712.5	131 (56.22%)	18 (7.72%)	516 (221.45%)
Asparagine	260.0	44 (53.01%)	11 (20.75%)	194 (233.73%)
Aspartate	287.5	53 (67.94%)	5 (6.84%)	208 (284.93%)
Cysteine	229.5	41 (59.42%)	4 (5.79%)	168 (243.47%)
Glutamate	304.5	57 (73.07%)	9 (11.53%)	219 (280.76%)
Glutamine	292.0	50 (56.81%)	5 (5.68%)	217 (246.59%)
Glycine	641.5	115 (65.34%)	17 (9.65%)	469 (266.47%)
Histidine	197.0	32 (52.45%)	7 (11.47%)	149 (244.26%)
Isoleucine	453.6	97 (85.08%)	6 (5.26%)	308 (270.17%)
Leucine	1081.5	193 (61.66%)	25 (7.98%)	792 (253.03%)
Lysine	372.5	67 (56.77%)	9 (7.62%)	272 (230.50%)
Methionine	598.0	108 (59.34%)	19 (10.43%)	436 (239.56%)
Phenylalanine	276.5	47 (56.62%)	8 (9.63%)	206 (248.19%)
Proline	699.5	133 (70.74%)	16 (8.51%)	500 (265.95%)
Serine	788.5	139 (55.60%)	23 (9.2%)	580 (232.00%)
Threonine	596.5	107 (57.83%)	15 (8.10%)	436 (235.67%)
Tryptophan	284.0	54 (70.12%)	5 (6.49%)	203 (263.63%)
Tyrosine	212.0	38 (57.57%)	6 (9.09%)	155 (234.84%)
Valine	485.5	85 (57.82%)	19 (12.92%)	358 (243.53%)

*Highest transition/transversion bias was found in tRNA^Trp^ whereas the lowest was found in tRNA^Ile^*.

**Table 6:** Transition/transversion bias of cyanobacterial tRNAs.

**tRNA**	**k1 (Purines)**	**k2 (Pyrimidines)**	**R (Transition/Transversion Bias)**	**No. of sequences Studied**
Alanine	1.494	1.515	0.732	258
Arginine	2.607	2.449	1.207	255
Asparagine	3.389	3.268	1.618	90
Aspartate	6.971	10.501	4.131	84
Cysteine	4.699	1.87	1.65	78
Glutamate	3.951	2.82	1.626	87
Glutamine	1.626	4.461	1.499	94
Glycine	3.2	3.426	1.631	192
Histidine	2.832	2.25	1.199	68
Isoleucine	0.231	1.512	0.39	124
Leucine	1.845	2.114	0.964	343
Lysine	3.149	3.386	1.623	126
Methionine	1.805	3.28	1.231	202
Phenylalanine	1.619	1.661	0.811	89
Proline	3.708	4.158	1.825	204
Threonine	3.133	3.453	1.618	202
Tryptophan	26.77	7.30	8.409	83
Tyrosine	5.114	10.015	3.658	76
Serine	0.914	1.82	0.654	271
Valine	1.959	2.169	1.018	159

*Result showed loss of cyanobacterial tRNA gene predominate the duplication and conditional duplication event*.

The authors apologize for this error and state that this does not change the scientific conclusions of the article in any way. The original article has been updated.

